# Supplementary material for: iPAR: A framework for modelling and inferring information about disease spread when the populations at risk are unknown
Source: PLoS Comput Biol. 2025 Jun 16;21(6):e1012622. doi: 10.1371/journal.pcbi.1012622 (PMC12204632; doi:10.1371/journal.pcbi.1012622)
Supplement: S1 Appendix — (DOCX) [file pcbi.1012622.s001.docx]

**Appendix 1: Kernel normalisation**

The transmission kernel described in the main text is a truncated power law $K\left( d \right)=d^{-2\lambda}I(d<d_{\text{max}})/m(\lambda)$ where $m(\lambda)$ is a normalisation constant introduced in [1] and defined as follows.

$$m\left( \lambda\right)=\sum_{(x,y)\in\mathbb{Z}^{2}\backslash\{(0.0)\}} \left( x^{2}+y^{2} \right)^{-\lambda}I(x^{2}+y^{2}<d_{\text{max}}^{2})$$

Intuitively, $m(\lambda)$ is the sum of the force of infection described by the unnormalized kernel, given a single source of infection at coordinates $(0,0)$ and susceptible units arranged on a two-dimensional integer lattice. If we divide the unnormalized kernel by this sum, it means that changing $\lambda$ will only change the *spatial distribution* of the force of infection arising from a single infectious unit, rather than changing the *total* force of infection on the surrounding landscape. This normalisation leads to better separation of the effect of $\lambda$ from the effect of the overall rate of infection $\rho$. It does not lead to any changes in the parameters except for the value of $\rho$. It implicitly assumes that the patches of the model are arranged on an integer lattice. If this assumption is violated then the normalisation will work less well. There will also be edge effects near the boundaries of the modelled region.

**References**

1. Cook, Alex, Glenn Marion, Adam Butler, and Gavin Gibson. 2007. “Bayesian Inference for the Spatio-Temporal Invasion of Alien Species.” Bulletin of Mathematical Biology 69 (6): 2005–25. https://doi.org/10.1007/s11538-007-9202-4.
