# Supplementary material for: iPAR: A framework for modelling and inferring information about disease spread when the populations at risk are unknown
Source: PLoS Comput Biol. 2025 Jun 16;21(6):e1012622. doi: 10.1371/journal.pcbi.1012622 (PMC12204632; doi:10.1371/journal.pcbi.1012622)
Supplement: S2 Appendix — (DOCX) [file pcbi.1012622.s002.docx]

**Appendix 2: Prior distributions**

The prior distributions for the model parameters were taken to be uninformative, and were generally chosen to be uniform distributions over their chosen supports. Most parameters had independent prior distributions, with the exception of the two simplex parameters where it was essential to allow for dependence between the simplex components. To fit the iPAR model, whether time-varying or constant-in-time, to the Estonian data in the case study, we chose the following prior distributions.

| **Parameter** | **Prior distribution** |
| --- | --- |
| $\sigma$, $\gamma$ | Uniform on the simplex $S^{6}$ |
| $\lambda$ | Uniform on the interval $(0,10)$ |
| $\rho$ | Uniform on the interval $(0,10000)$ |
| $\varepsilon$ | Uniform on the interval $(0,10)$ |
| $h_{i}$ | Uniform on the interval $(0.1,100)$ |
