# Supplementary material for: iPAR: A framework for modelling and inferring information about disease spread when the populations at risk are unknown
Source: PLoS Comput Biol. 2025 Jun 16;21(6):e1012622. doi: 10.1371/journal.pcbi.1012622 (PMC12204632; doi:10.1371/journal.pcbi.1012622)
Supplement: S3 Appendix — (DOCX) [file pcbi.1012622.s003.docx]

**Appendix 3: Likelihood and sampling algorithm**

**Likelihood**

The likelihood for the varying-in-time model is

$$P\left( \mathcal{T,U} | \theta\right)=\left[ \prod_{\{i:0<T_{i}\leq N\}} \left\{ s_{i}h(U_{i})\left( \varepsilon+\rho\sum_{\{j:U_{j}<U_{i}\}} t_{j}K(d_{ij}) \right) \right\}\times\text{exp}\left\{ -s_{i}\left( \varepsilon\int_{0}^{U_{i}} h\left( t \right)dt+\rho\sum_{\{j:U_{j}<U_{i}\}} t_{j}K(d_{ij})\int_{U_{j}}^{U_{i}} h\left( t \right)dt \right) \right\} \right]\times\prod_{\{i:T_{i}=N+1\}} \text{exp}\left\{ -s_{i}\left( \varepsilon\int_{0}^{T} h\left( t \right)dt+\rho\sum_{\{j:T_{j}\leq N\}} t_{j}K(d_{ij})\int_{U_{j}}^{T} h\left( t \right)dt \right) \right\}$$

The above expression is conditioned on the history of transmission prior to time $t=0$and written under the assumption that the time category observations $\mathcal{T}$ are consistent with the given infection times $\mathcal{U}$. The second product represents the probability that patches never observed to be infected escape infection up to time $T.$ On the other hand, the first product is over patches observed as infected in the time interval $[0,T]$ and represents the probability of their infection at the latent infection times $\mathcal{U}$. Proposed changes to $\mathcal{U}$ - made in the sampling algorithm described below – must therefore be consistent with $\mathcal{T}$. The values of the time-varying function, $h_{i}$, are regarded as parameters to be estimated from the data and they are appended to the parameter set $\theta$. The corresponding likelihood for the constant-in-time model is obtained by setting the function $h$ equal to $1$ for all times $t$.

**Sampling algorithm**

Here we provide further details of the Markov chain Monte Carlo (MCMC) algorithm used to obtain samples from the joint posterior distribution $P(\theta, \mathcal{U|T)}$ of the model parameters $\theta$ and the actual unobserved patch infection times $\mathcal{U}$, where $\mathcal{T}$ denotes the set of interval-censored patch infection times which we assume are provided to us as data. The algorithm initialises and simulates a Markov chain whose stationary distribution is the posterior distribution. At each iteration of the algorithm, updates are proposed to each parameter and infection time in turn. A more precise description follows, in which the superscript $(m)$ indicates the value of a quantity at the $m$’th iteration of the algorithm. We use the same notation as used in Methods.

**Initialisation**

Initialise parameters with values that are arbitrary but also consistent with the selected prior distributions. For a patch $i$, let $T_{i}\epsilon\{0,1,2,\ldots,N+1\}$ denote the ‘time category’ into which the patch falls. Then, for $1\leq T_{i}\leq N$, we initialise the infection time of the patch uniformly at random on the time interval $(t_{j-1},t_{j}]$. If $T_{i}=0$ then the patch is taken to be infected at the beginning of the modelled time period. If $T_{i}=N+1$ then the patch is taken to be uninfected at the end of the modelled time period.

**Iteration**

Repeat the following sequence a large number of times:

(a) Evaluate the current likelihood. In the manuscript this is denoted $P\left( \mathcal{T,U} | \theta\right)$ but for simplicity we denote it here by $L$. Propose a new value for the transmission parameter $\lambda$ by sampling from the proposal distribution $q(\lambda^{\left( m \right)},\lambda^{*})$. Specifically, we take $\lambda^{*}\sim N(\lambda^{\left( m \right)},\sigma_{\lambda}^{2})$ which is a normal distribution centred on the current value of the parameter. Recalculate the likelihood with the proposed parameter value, to give a new likelihood $L^{*}$. The proposed change is accepted with Metropolis-Hastings acceptance probability

$$\min\left( 1,\frac{L^{*}P(\lambda^{*})q(\lambda^{*},\lambda^{\left( m \right)})}{LP(\lambda^{\left( m \right)})q(\lambda^{\left( m \right)},\lambda^{*})} \right)$$

In this case, we take $\lambda^{\left( m+1 \right)}=\lambda^{*}$ and the likelihood is updated to its new value $L^{*}$. If the change is rejected then we take $\lambda^{\left( m+1 \right)}=\lambda^{\left( m \right)}$ and the likelihood remains the same. Note that, because the proposal distribution is symmetric, the two proposal factors in the acceptance probability cancel, which simplifies the calculations.

(b) Repeat (a) for the other model parameters in turn: overall rate $\rho$, background rate $\varepsilon$, time varying parameters $h_{i}$ (for the varying-in-time model), the susceptibility parameters $\sigma_{k}^{'}$ and the infectivity parameters $\gamma_{k}^{'}$.

(c) Sample the simplex parameters $\sigma$ and then $\gamma$. The components of $\sigma$ are constrained to sum to 1, so we can’t sample the components independently in the same way that, for example, the susceptibility parameters $\sigma_{k}^{'}$ can be sampled independently, one at a time. Instead, we use a joint proposal distribution $q(\sigma^{\left( m \right)},\sigma^{*})$. Specifically, we take

$$\sigma^{*}\sim\mathrm{Dir} \left( \delta+\alpha_{\sigma}\sigma_{1}^{(m)},\delta+\alpha_{\sigma}\sigma_{2}^{(m)},\cdots,\delta+\alpha_{\sigma}\sigma_{L}^{(m)} \right)$$

This is a Dirichlet distribution with a specified set of concentration parameters. It is the analogue, for parameters on the simplex, of the Gaussian random walk proposal used in (a) for parameters defined on real intervals. The acceptance probability and other aspects of the procedure are the same as in step (a). However, in this case the proposal distribution is not symmetric, so the two proposal factors in the acceptance probability do not cancel. The sampling process is then repeated for the other simplex parameter $\gamma$.

(d) Sample the unobserved patch infection times $U_{i}$, taking each one in turn. This is carried out in the same way as in (a), with a normal proposal density $U_{i}^{*}\sim N(U_{i}^{(m)},\sigma_{i}^{2})$ and acceptance probability

$$\min\left( 1,\frac{L^{*}q(U_{i}^{*},U_{i}^{(m)})}{Lq(U_{i}^{(m)},U_{i}^{*})} \right)$$

**Optimisation**

The first 10000 iterations of each Markov chain are discarded as ‘burnin’. During the burnin phase, the proposal distribution parameters such as $\sigma_{\lambda}^{2}$, $\alpha_{\sigma}$ and $\sigma_{i}^{2}$ are adapted to ensure that the acceptance probabilities are not too large or small. This is done in a similar manner to that described in [1]. The parameter $\delta$ in the Dirichlet proposal was fixed at $1$, a value obtained by trial and error; it was not adapted in the same way as the other proposal distribution parameters. Its purpose is to stabilise the algorithm, in particular to prevent proposed values from getting too close to the boundary of the simplex.

When the likelihood is recalculated during the MCMC, only the parts of the likelihood that might actually change are computed. This ensures that the computation time is kept to a minimum.

**MCMC convergence**

Typically, five chains are run in order to fit the model. The Gelman-Rubin convergence test is used to assess convergence, along with inspection of trace plots, autocorrelation, between-parameter correlation and estimation of the effective sample size.

**References**

1. Pooley, Christopher M., Glenn Marion, Stephen C. Bishop, Richard I. Bailey, and Andrea B. Doeschl-Wilson. 2020. “Estimating Individuals’ Genetic and Non-Genetic Effects Underlying Infectious Disease Transmission from Temporal Epidemic Data.” *PLoS Computational Biology* 16 (12 December). https://doi.org/10.1371/journal.pcbi.1008447.
