# Supplementary material for: iPAR: A framework for modelling and inferring information about disease spread when the populations at risk are unknown
Source: PLoS Comput Biol. 2025 Jun 16;21(6):e1012622. doi: 10.1371/journal.pcbi.1012622 (PMC12204632; doi:10.1371/journal.pcbi.1012622)
Supplement: S4 Appendix — (DOCX) [file pcbi.1012622.s004.docx]

**Appendix 4: DIC computation and values**

In this appendix we use the same notation as in [1]. In that paper, the DIC metric ${DIC}_{1}$ is defined by the following formula

|  | ${DIC}_{1}=-4\mathbb{E}_{\theta,X}\left[ \log\left( f\left( y,X \vert\theta\right) \right)\vert y \right]+2\mathbb{E}_{X}[\log\left( f(y,X\vert\mathbb{E}_{\theta}\left[ \theta\vert y,X \right]) \right)\vert y]$ | ( 1 ) |
| --- | --- | --- |

Here, $f$ is the complete/full likelihood, $y$ represents the observed data, $X$ represents the unobserved data, while $\theta$ is the model parameters. This metric corresponds to ${DIC}_{4}$ in [2]. For each value of $X$, the second term requires evaluation of a posterior expectation (conditional on that value of $X$), so ${DIC}_{1}$ is quite expensive to compute if there is no closed form available, or if the dimension of $\theta$ is high. However, inspection of the supplementary material of [1] shows that, in fact, the following metric was computed

|  | ${DIC}_{1}=-4\mathbb{E}_{\theta,X}\left[ \log\left( f\left( y,X \vert\theta\right) \right)\vert y \right]+2\mathbb{E}_{X}[\log\left( f(y,X\vert\hat{\theta}(y)) \right)\vert y]$ | ( 2 ) |
| --- | --- | --- |

Here $\hat{\theta}(y)$ is the posterior expectation of the parameter vector given the data. This metric is a version of ${DIC}_{6}$ in [2]. It is this latter metric that has been computed for the fitted models in the manuscript, but with $\hat{\theta}(y)$ being the posterior median rather than expectation. When we refer to ${DIC}_{1}$ in the manuscript, it is this second definition that we are assuming.

The computation of ${DIC}_{1}$ is an additional task which can be carried out after the MCMC algorithm has been completed. During the MCMC we store the loglikelihood values obtained at each iteration. Averaging these stored loglikelihood values gives the first expectation in (2) above. The second expectation requires a bit more work. First, we compute the posterior medians $\hat{\theta}(y)$. Then, at each iteration/sample we compute the loglikelihood evaluated at $\hat{\theta}(y)$ and the ‘current’ set of latent variables. Averaging these loglikelihoods over all iterations gives the second expectation in (2). Note that the samples can be thinned before carrying out these computations, which substantially reduces computation time.

$\boldsymbol{DIC}_{\boldsymbol{1}}$ **values for Estimation of temporal trends in transmission in Results**

More context regarding the simulated scenarios listed below is provided in the main text. The lowest ${DIC}_{1}$ for each scenario, which is the ${DIC}_{1}$ of the favoured model, is highlighted in bold.

| **Scenario** | **Constant-in-time model** | **Varying-in-time model** |
| --- | --- | --- |
| 1 (linear decrease) | 2908 | **2874** |
| 2 (constant) | **2882** | 2886 |
| 3 (sudden drop off) | 2961 | **2935** |

$\boldsymbol{DIC}_{\boldsymbol{1}}$ **values for Parameter estimates in Case study**

More context regarding the data used to fit these models is provided in Case study. The lowest ${DIC}_{1}$, which is the ${DIC}_{1}$ of the favoured model, is highlighted in bold.

| **Constant-in-time model** | **Varying-in-time model** |
| --- | --- |
| 3203 | **2987** |

**References**

1. Gamado, Kokouvi, Glenn Marion, and Thibaud Porphyre. 2017. “Data-Driven Risk Assessment from Small Scale Epidemics: Estimation and Model Choice for Spatio-Temporal Data with Application to a Classical Swine Fever Outbreak.” *Frontiers in Veterinary Science* 4 (FEB). https://doi.org/10.3389/fvets.2017.00016.
2. Celeux, G, F Forbes, C P Robert, and D M Titterington. 2006. “Deviance Information Criteria for Missing Data Models.” *Bayesian Analysis*. Vol. 1.
