# Supplementary material for: iPAR: A framework for modelling and inferring information about disease spread when the populations at risk are unknown
Source: PLoS Comput Biol. 2025 Jun 16;21(6):e1012622. doi: 10.1371/journal.pcbi.1012622 (PMC12204632; doi:10.1371/journal.pcbi.1012622)
Supplement: S5 Appendix — (DOCX) [file pcbi.1012622.s005.docx]

**Appendix 5: the simplex and the isometric logratio transformation**

The disease transmission model described in the manuscript uses parameters for susceptibility and infectivity that are constrained to the simplex $S^{D}=\{\sigma\in\mathbb{R}^{D}: \sigma_{k}>0 \forall k, \sum_{k=1}^{D} \sigma_{k}=1\}$, where $D=6$ in the case study. Estimating a parameter constrained to the simplex can be challenging due to counterintuitive effects. Two possible effects are the following:

1. It can be difficult to estimate parameter values that are on or close to the boundary of parameter space, and credible intervals as commonly constructed may not contain the true parameter value. So, we can expect difficulties if a simplex parameter is close to the boundary of the simplex. One approach that can sometimes help is the $\text{ilr}$ transform, which we discuss below.
2. The prior distribution on the simplex is assumed to be uniform, which implies that the prior mean is the centroid of the simplex. If the data are not very informative then simplex parameter estimates are likely to be drawn towards the centroid somewhat.

Below, we consider parameter estimation for a simple multivariate normal model on the simplex. Our purpose here is to highlight that the above two effects can be relevant even for very simple models. Then, we consider the above two effects for the disease transmission model used in this manuscript.

**The isometric logratio transform**

The isometric logratio transform $\text{ilr}:S^{D}\to\mathbb{R}^{D-1}$ is defined by

$${\text{ilr}\text{(σ)}}_{i}=\sqrt{\frac{i}{i+1}}\text{ln}\left( \frac{G(\sigma_{1},\ldots,\sigma_{i})}{\sigma_{i+1}} \right)$$

where $G$ denotes the geometric mean and $1\leq i\leq D-1$ [1]. This transform is helpful because it maps simplex parameters to more familiar real vector-valued parameters. On the other hand, the components of $\text{ilr}\text{(σ)}$ may not be easy to interpret in terms of the components of $\text{σ}$.

**The multivariate normal simplex model**

The model is as follows. It has a single parameter $\mu\in S^{D}$. Observed data are assumed to be independent samples from the normal on $S^{D}$ distribution $N_{S}^{D}(\text{ilr}(\mu),\Sigma)$, which was introduced by [2]. This distribution is essentially a multivariate normal distribution on the simplex and has density

$$N_{S}^{D}\left( p;\text{ilr}(\mu),\Sigma\right)={(2\pi)}^{-\frac{D-1}{2}}\left| \Sigma\right|^{-\frac{1}{2}}\text{exp}\left( -\frac{1}{2}\left( \text{ilr}(p)-\text{ilr}(\mu) \right)^{T}\Sigma^{-1}(\text{ilr}(p)-\text{ilr}(\mu)) \right)$$

Where, for simplicity, the covariance matrix $\Sigma$ is assumed known and equal to $\sigma^{2}I$. In summary, the observed data points are $y_{i}\sim N_{S}^{D}(\text{ilr}(\mu),\sigma^{2}I)$ for $1\leq i\leq n$.

Suppose also that we assume a spherically symmetric prior for $\mu$ of the form $N_{S}^{D}(0,\sigma_{prior}^{2}I)$. The posterior distribution for $\mu$ can then be computed analytically in the same way as for multivariate normal data (see e.g. [3]) to obtain $p\left( \mu| y \right)=N_{S}^{D}(\frac{\sigma^{-2}\sum_{i=1}^{n} \text{ilr}\left( y_{i} \right)}{{n\sigma}^{-2}+\sigma_{prior}^{-2}},\frac{1}{{n\sigma}^{-2}+\sigma_{prior}^{-2}}I)$. The mean vector is a convex combination of the prior mean in real space (zero) and $\frac{1}{n}\sum_{i=1}^{n} \text{ilr}\left( y_{i} \right)$, as expected. Thus, even if $\frac{1}{n}\sum_{i=1}^{n} \text{ilr}\left( y_{i} \right)$ is close to $\text{ilr}(\mu)$, the mean of the posterior will be drawn somewhat towards zero by the influence of the prior. This is to be expected and is not surprising. However, it is perhaps less obvious what this means within the simplex: even if $\frac{1}{n}\sum_{i=1}^{n} \text{ilr}\left( y_{i} \right)=\text{ ilr}(\mu)$, the posterior mode is drawn away from $\mu$ and towards the centroid of the simplex. More precisely, the posterior mode will be of the form $\mathcal{C}\left( \mu^{\alpha} \right)$ where $\alpha=\frac{n\sigma^{-2}}{{n\sigma}^{-2}+\sigma_{prior}^{-2}}$, $\mathcal{C}$ denotes the closure operator which divides a vector by the sum of its components, and the power operation is applied to each component of $\mu$. In particular, if $i$ denotes the index of the largest component of $\mu$, then it can be seen that ${\mathcal{C}\left( \mu^{\alpha} \right)}_{i}<\mu_{i}$. So the largest component of the parameter vector $\mu$ will be drawn downwards, especially when the number of data points is very small.

The following example illustrates the model in the case $D=3$, with $n=1$ representing a scenario where the available data are very limited. Data are simulated from the model 100000 times. For each dataset, an estimate of $\mu$ is obtained. These estimates are plotted in Figure A1 and Figure A2. There is clearly a tendency for the largest component of of $\mu$ to be drawn towards the centroid of the simplex.


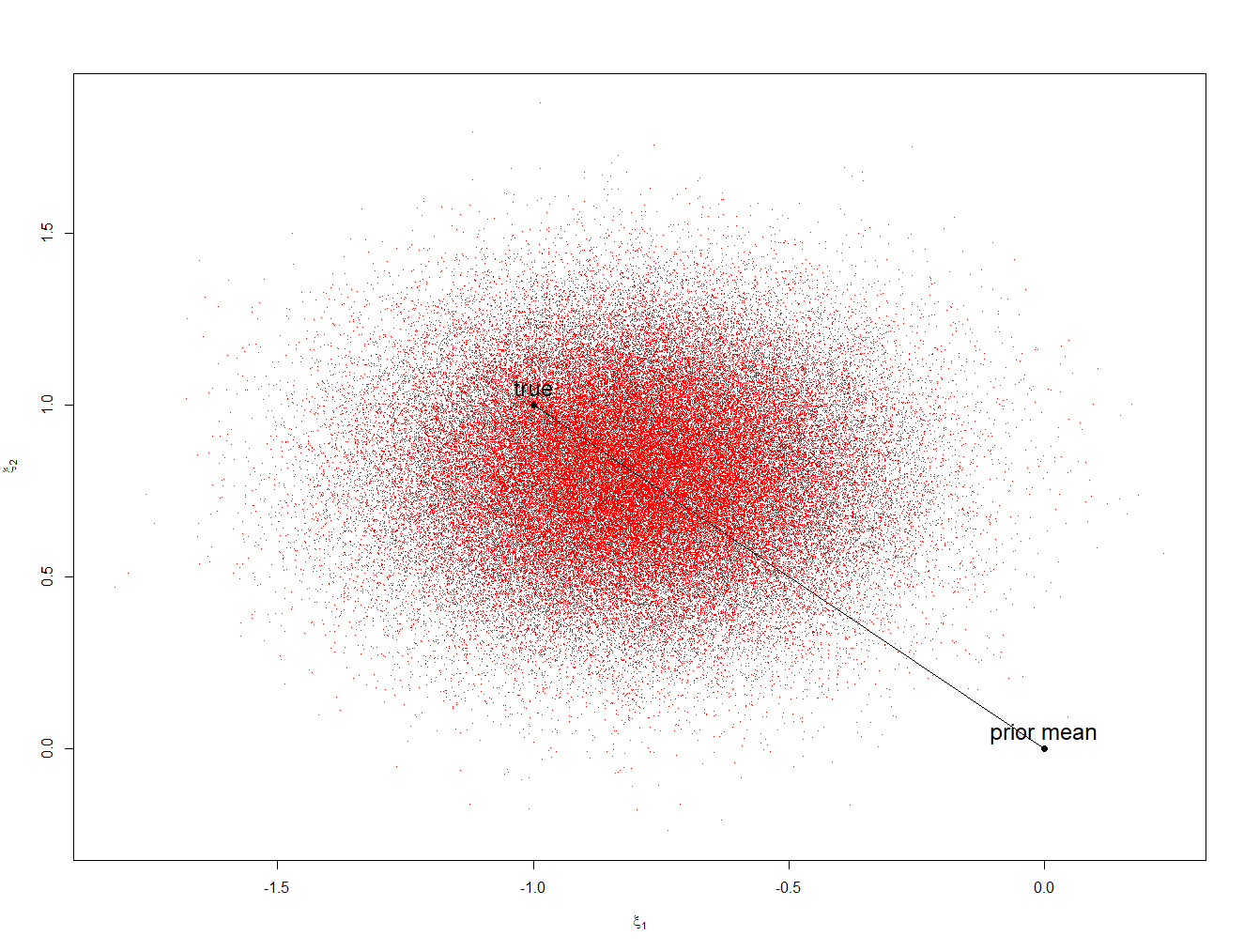


Figure A1. Plot of 100000 estimates (posterior mode) of $\mu$ in real space. The true value of $\mu$ is denoted by ‘true’. The straight line connects the true value of $\mu$ to the prior mean.


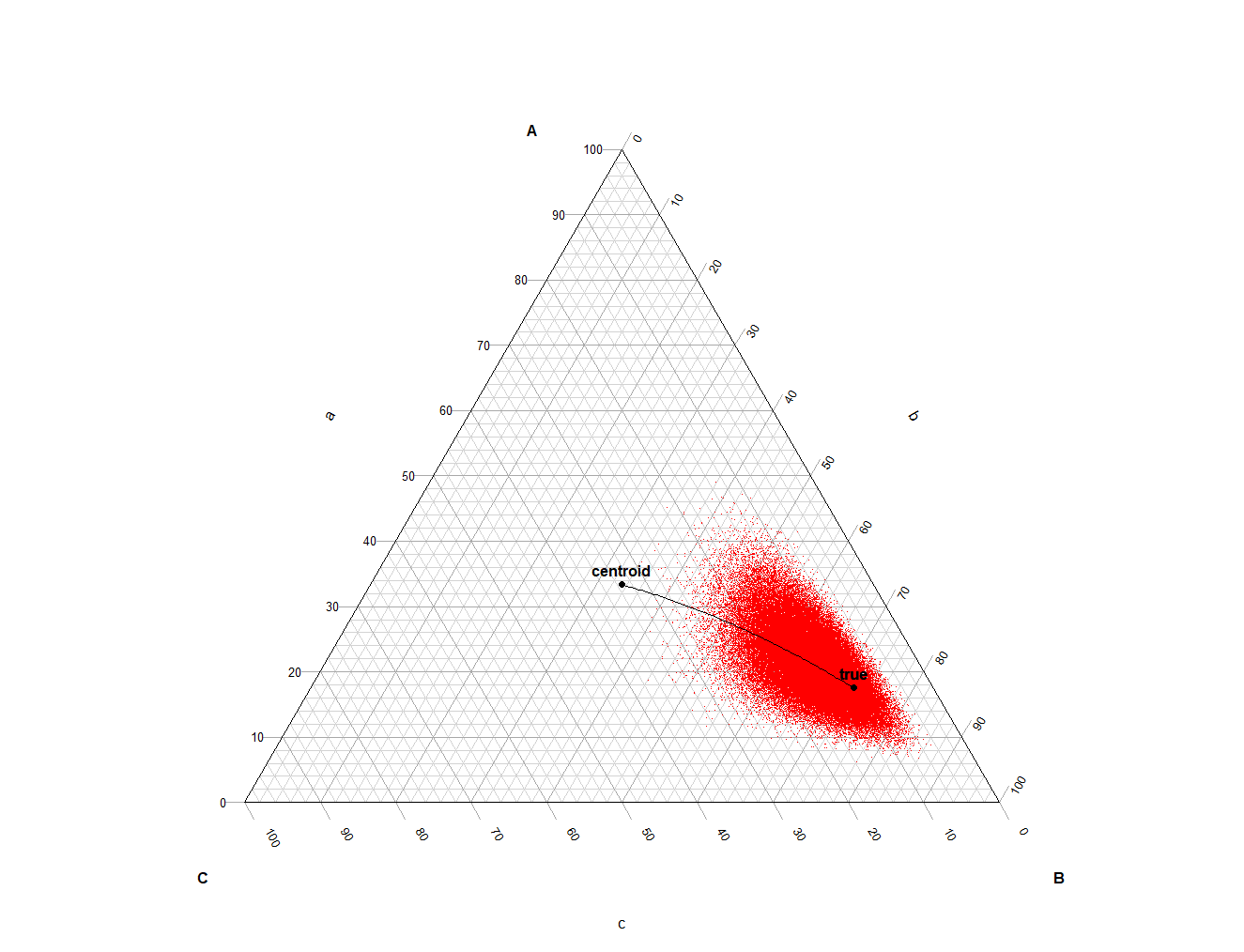


Figure A2. Plot of 100000 estimates (posterior mode) of $\mu$ in the simplex. The true value of $\mu$ is denoted by ‘true’. The straight line (in terms of the simplex geometry) connects the true value of $\mu$ to the centroid of the simplex.

The model setup here is artificial and we have a made a number of unrealistic assumptions. Nevertheless, it is hoped that this shows why it is common that the largest component of a simplex-valued parameter tends to be underestimated.

**The disease transmission model**

We present here an example of inference based on data simulated from the disease transmission model presented in the manuscript, except that the dimension $D$ of the simplex parameters has been reduced to $D=3$. This is achieved by amalgamating some of the land use categories. The reason for doing this is to make it easier to visualise the results of the inference. However, it should be borne in mind that reducing $D$ may also reduce some of the previously discussed effects on parameter estimation.

Figures A3 and A4 show the infectivity parameter posterior samples obtained following inference from a set of simulated data with $D=3$. Comparing the two figures shows that, while of value, the univariate credible intervals do not give a complete picture of the posterior distribution.


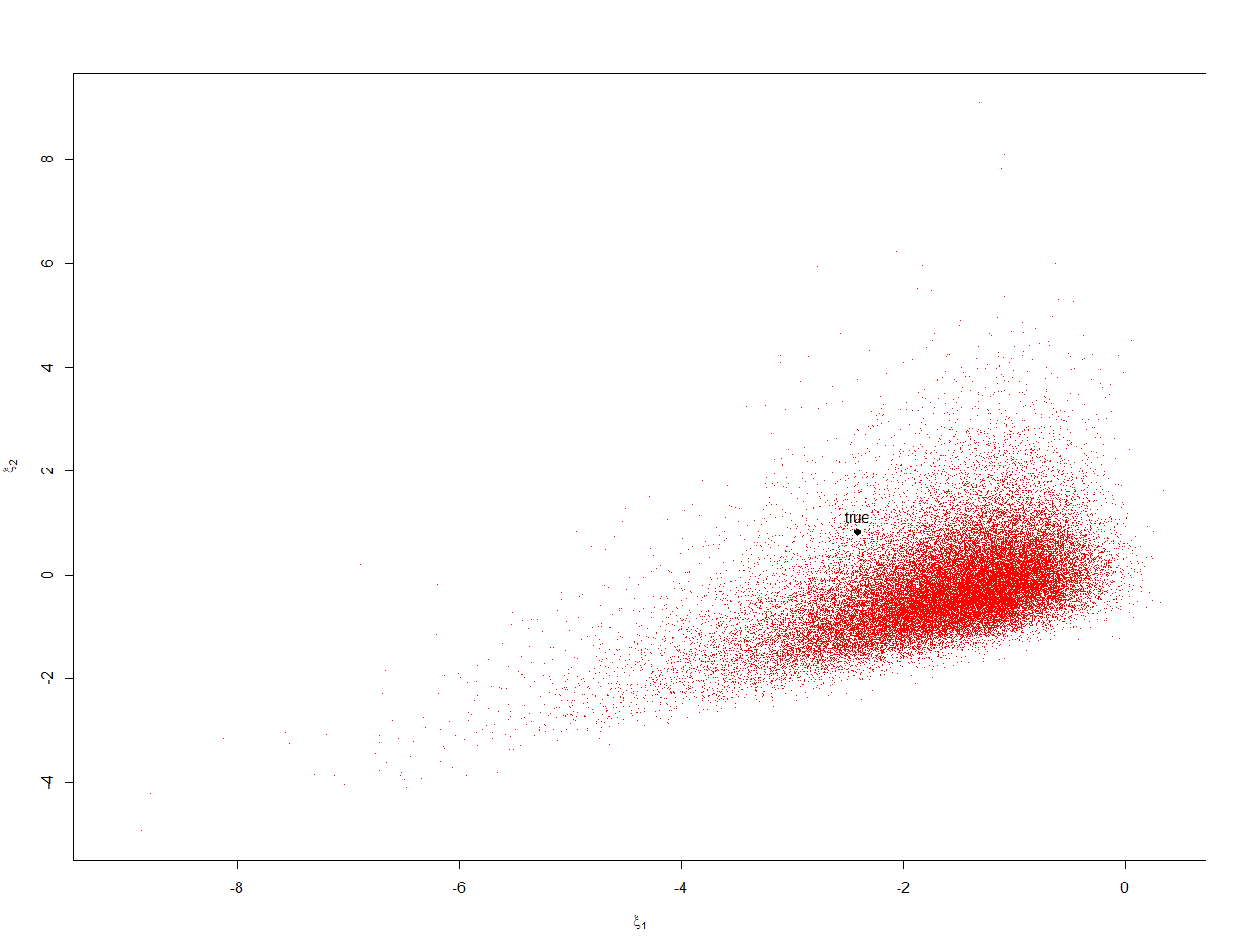


Figure A3. Posterior samples of the infectivity vector in real space, plotted along with the true value. Both univariate 95% credible intervals contain the true parameter.


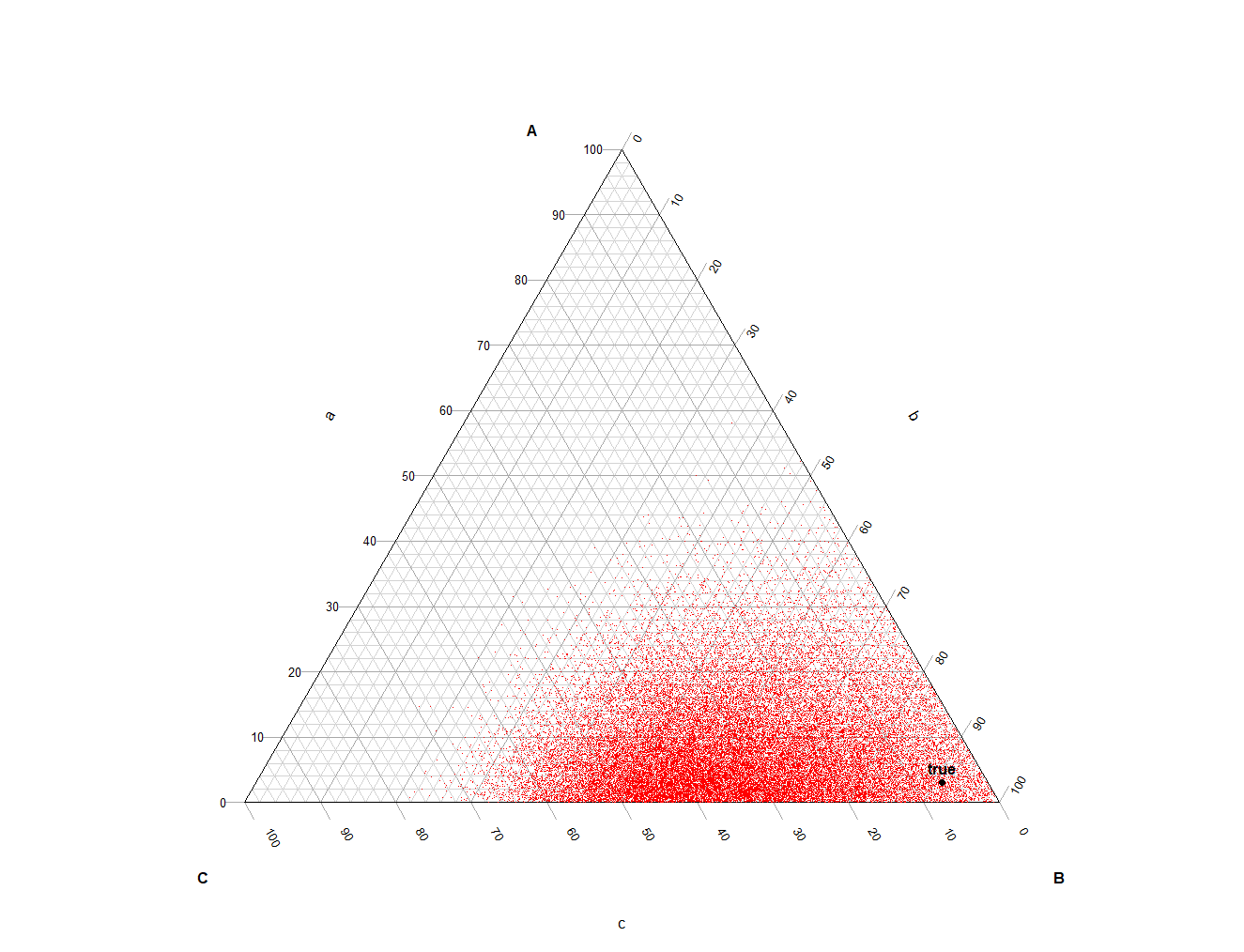


Figure A4. Posterior samples of the infectivity vector in the simplex, plotted along with the true value. The univariate 95% credible intervals for A and C contain the corresponding true values, while the 95% credible interval for B (0.35, 0.89) does not.

Next, we present corresponding results for the disease transmission model with $D=6$ (Figures A5, A6). In this case, we cannot visualise all dimensions simultaneously. Nevertheless, it appears that the true parameter value is better estimated when viewed in real space than when viewed in simplex space.
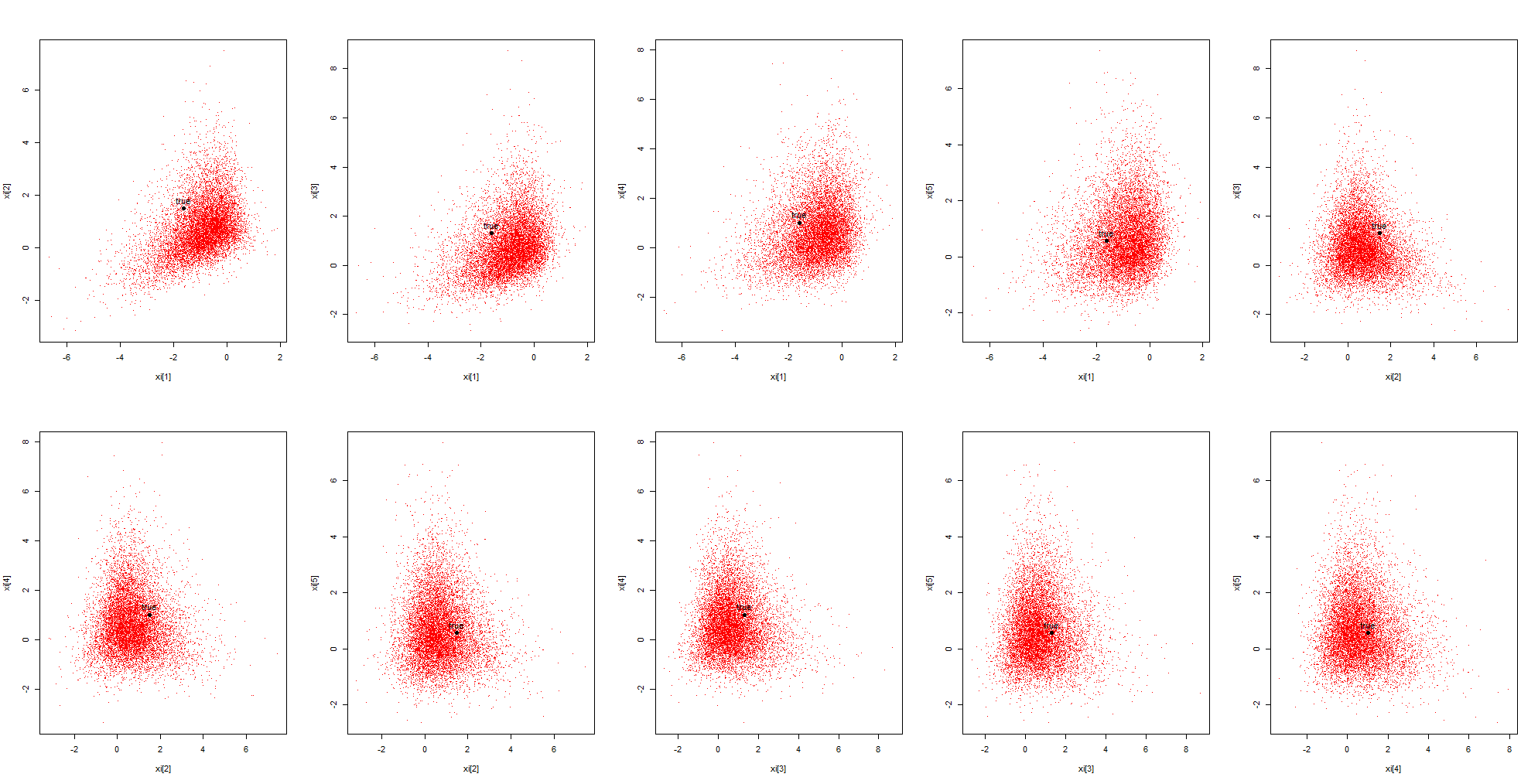


Figure A5. Posterior samples of the infectivity vector in real space, plotted along with the true value. All univariate 95% credible intervals contain the true parameter.


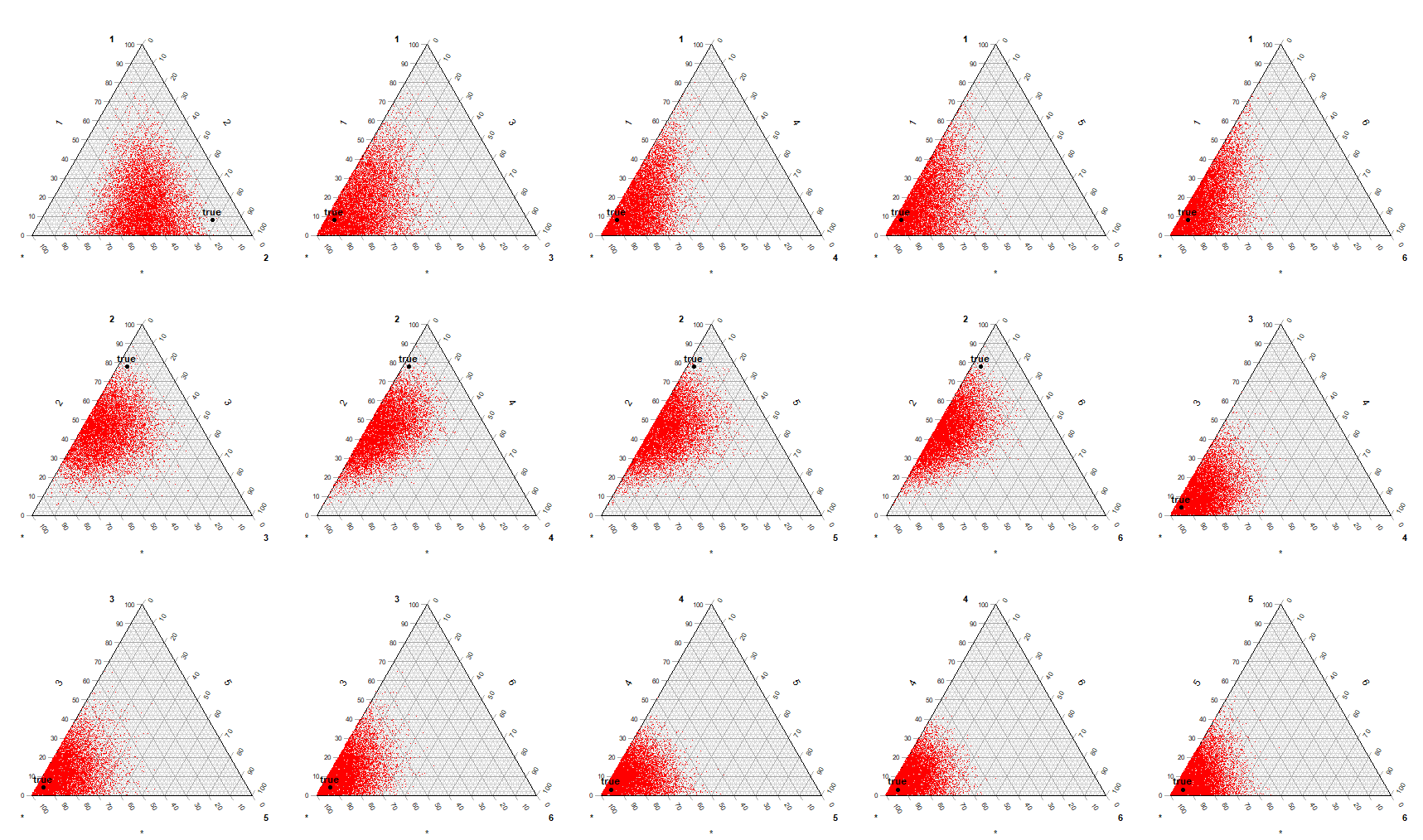


Figure A6. Posterior samples of the infectivity vector in the simplex, plotted along with the true value. The univariate 95% credible intervals all contain the true parameter value, with the exception of parameter 2.

**References**

1. Egozcue, Juan José, Vera Pawlowsky-Glahn, Glòria Mateu-Figueras, and Carles Barcelo-Vidal. Isometric logratio transformations for compositional data analysis. Mathematical geology 35, no. 3 (2003): 279-300.
2. Mateu-Figueras, G., Pawlowsky-Glahn, V. and Egozcue, J. J. (2013). The normal distribution in some constrained sample spaces. *SORT*, 37, 29–56.
3. A. Gelman, J. B. Carlin, H. S. Stern, and D. B. Rubin (2003). Bayesian Data Analysis. Chapman & Hall/CRC, London, 2nd edition.
