# Supplementary material for: iPAR: A framework for modelling and inferring information about disease spread when the populations at risk are unknown
Source: PLoS Comput Biol. 2025 Jun 16;21(6):e1012622. doi: 10.1371/journal.pcbi.1012622 (PMC12204632; doi:10.1371/journal.pcbi.1012622)
Supplement: S6 Appendix — (DOCX) [file pcbi.1012622.s006.docx]

**Appendix 6: additional figures for Estimation of key epidemiological parameters in Results**

This Appendix provides additional figures for the simulation study in Estimation of key epidemiological parameters in Results. The plots show true versus inferred parameters for $\xi=\text{ilr(}\text{σ}\text{)}\text{∈}\mathbb{R}^{5}$ and $\eta=\text{ilr(}\text{γ}\text{)}\text{∈}\mathbb{R}^{5}$. Testing the iPAR modelling approach in Methods discusses the reason for using the $\text{ilr}$ transform on these parameters.


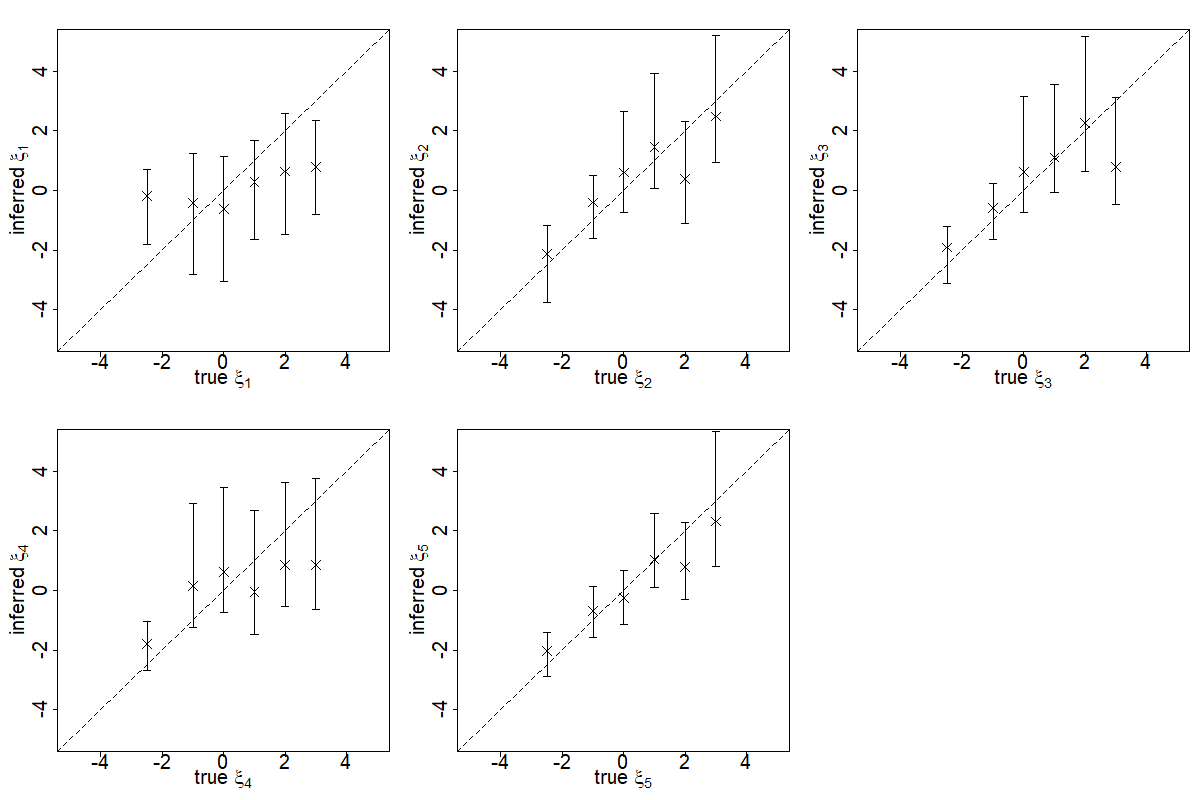


Figure A7. Assessment of the reliability of parameter inference for the constant-in-time iPAR model. Each panel corresponds to a parameter of the model. The ‘true’ value of the parameter is plotted against the posterior median (cross) and posterior 95% credible interval (vertical line). For comparison purposes we also superimpose the diagonal line representing perfect agreement between inferred and true values. This figure includes panels for all five components of $\xi$, the $\text{ilr}$ transformed susceptibility parameter.


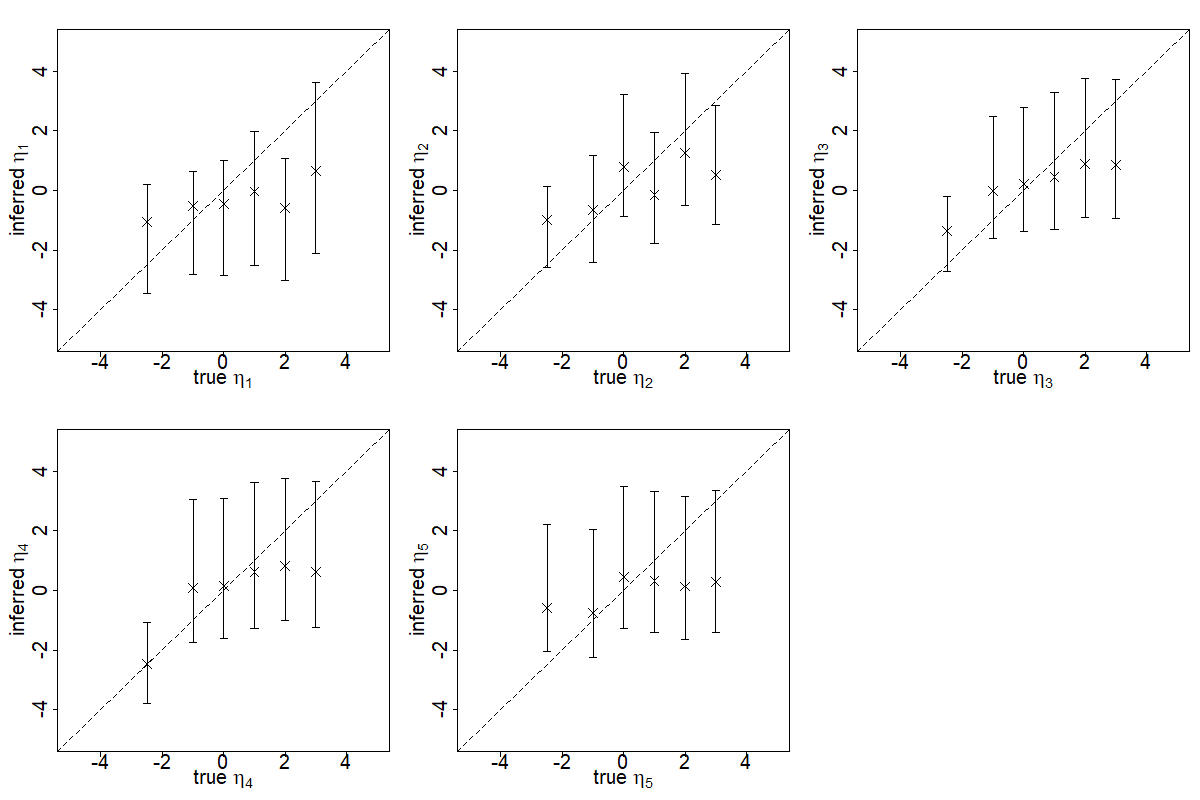


Figure A8. Assessment of the reliability of parameter inference for the constant-in-time iPAR model. Each panel corresponds to a parameter of the model. The ‘true’ value of the parameter is plotted against the posterior median (cross) and posterior 95% credible interval (vertical line). For comparison purposes we also superimpose the diagonal line representing perfect agreement between inferred and true values. This figure includes panels for all five components of $\eta$, the $\text{ilr}$ transformed infectivity parameter.
