# Supplementary material for: iPAR: A framework for modelling and inferring information about disease spread when the populations at risk are unknown
Source: PLoS Comput Biol. 2025 Jun 16;21(6):e1012622. doi: 10.1371/journal.pcbi.1012622 (PMC12204632; doi:10.1371/journal.pcbi.1012622)
Supplement: S7 Appendix — (DOCX) [file pcbi.1012622.s007.docx]

**Appendix 7: overestimation of the transmission rate** $\boldsymbol{\rho}$

In the simulation studies described in the main text, it was noted that there is typically slight overestimation of the ‘overall’ rate of infection $\rho$, which is proportional to the rate of transmission between two uniform patches (patches containing equal proportions of all land uses). Although we cannot make definitive conclusions regarding why this is the case for any specific model fit, we provide here some intuition as to why there might be overestimation.

First, it might seem surprising that an ‘overall’ rate of transmission could be difficult to estimate. However, it should be borne in mind that $\rho$ can be interpreted as the rate of transmission between two uniform patches. If some land uses are not well represented in the covariate data then there will be significant uncertainty in our knowledge of the rate of transmission between two uniform patches. A second issue is that the model aims to disentangle susceptibility and infectivity effects even with relatively poor data (disease cases have imprecise spatial and temporal coordinates). Given that it is known to be particularly difficult to estimate infectivity e.g. see Benefits of modelling spatial variation in susceptibility and infectivity in Results, and also [1], it is perhaps not surprising that estimation of $\rho$ is less straightforward than it might appear.

To be more precise, consider the rate of transmission between an infectious patch $j$ and a susceptible patch $i$ in the constant-in-time model.

$$r_{j\to i}=\rho\sum_{k=1}^{L} h_{i,k}\sigma_{k}\sum_{l=1}^{L} h_{j,l}\gamma_{l}$$

Here, $L=6$ for the models described in the main text, and we have ignored background transmission (which usually occurs only at a very low level) and the distance dependence (which usually seems well estimated). We further assume that the susceptibility parameter $\sigma$ is well estimated and that the infectivity $\gamma$ is poorly estimated and hence is drawn towards its prior. The outbreak data directly inform us about the rates $r_{j\to i}$. So, we might expect overestimation of $\rho$ – compensating for the poor estimation of $\gamma$ - when

$$\mathbb{E}_{\text{prior}}\left( \sum_{l=1}^{L} h_{j,l}\gamma_{l} \right)\leq\sum_{l=1}^{L} h_{j,l}\gamma_{l}$$

is satisfied on average across all patches $j$. The prior distribution of $\gamma$ is uniform on the simplex and its components sum to $1$, so the above condition, when averaged over all $j$, can be rewritten as

$$\frac{1}{L}\leq\sum_{l=1}^{L} h_{l}\gamma_{l}$$

Here, $h_{l}$ is the proportion of the modelled region (Estonia) occupied by land use category $l$. This condition essentially states that the land uses with highest infectivity are also the most common across the modelled landscape.

In the simulations of Estimation of temporal trends in transmission and Benefits of modelling spatial variation in susceptibility and infectivity in Results, this condition is always satisfied, and $\rho$ is consistently overestimated. In the simulations of Estimation of key epidemiological parameters in Results, the situation is more complex, with both overestimation and underestimation of $\rho$. We calculated $\Delta=\sum_{l=1}^{L} h_{l}\gamma_{l}-\frac{1}{L}$ for each of the 78 parameter combinations used in this latter section. Low/negative values of $\Delta$ seem to be strongly associated with underestimation of $\rho$ (Figure A9), which is in line with the informal argument presented above.


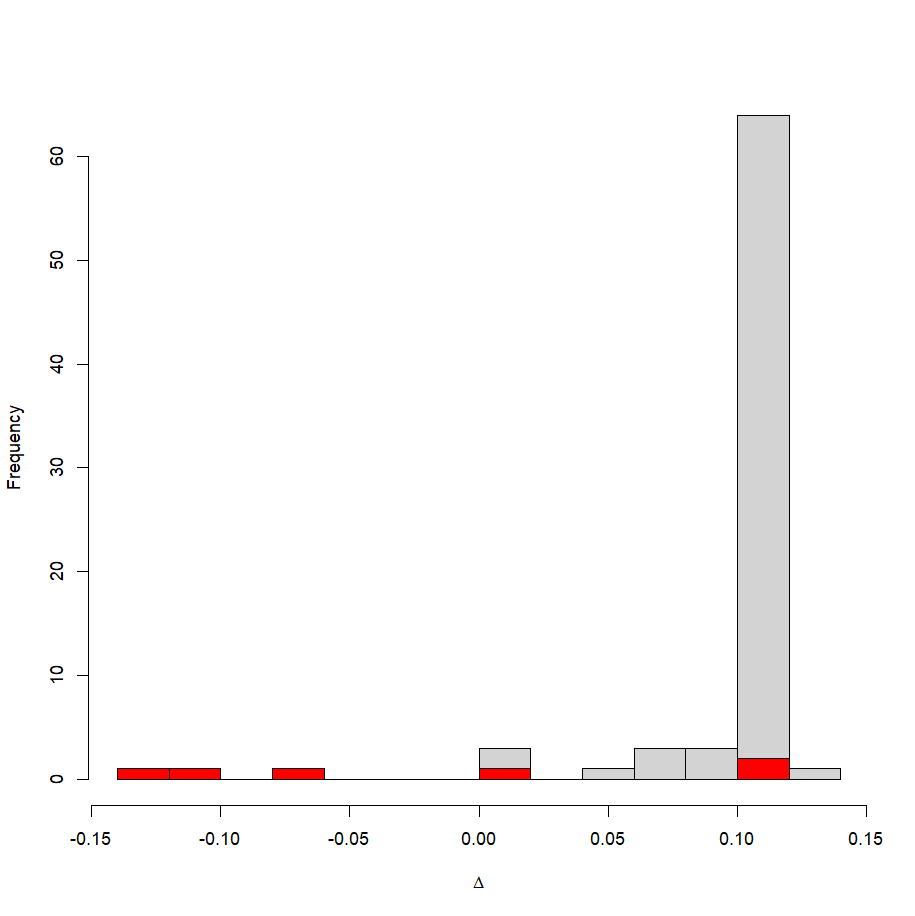


Figure A9. Histogram of $\Delta$ values for the 78 simulations (parameter combinations) in Estimation of key epidemiological parameters in Results. Grey denotes a simulation in which $\rho$ is overestimated, red denotes a simulation in which $\rho$ is underestimated. Note that the tall peak towards the right simply reflects the way in which the parameter combinations were selected; it does not have any other significance.

**References**

1. Pooley, Christopher M., Glenn Marion, Stephen C. Bishop, Richard I. Bailey, and Andrea B. Doeschl-Wilson. 2020. “Estimating Individuals’ Genetic and Non-Genetic Effects Underlying Infectious Disease Transmission from Temporal Epidemic Data.” *PLoS Computational Biology* 16 (12 December). https://doi.org/10.1371/journal.pcbi.1008447.
