# Supplementary material for: iPAR: A framework for modelling and inferring information about disease spread when the populations at risk are unknown
Source: PLoS Comput Biol. 2025 Jun 16;21(6):e1012622. doi: 10.1371/journal.pcbi.1012622 (PMC12204632; doi:10.1371/journal.pcbi.1012622)
Supplement: S8 Appendix — (DOCX) [file pcbi.1012622.s008.docx]

**Appendix 8: details of the setup for simulations in Benefits of modelling spatial variation in susceptibility and infectivity in Results**

This appendix details the parameter combinations used in the simulations for Benefits of modelling spatial variation in susceptibility and infectivity in Results. Four parameter combinations were used, corresponding to the four coloured rows in Table 1 of the main text. Either a wide or a moderate kernel was employed, together with a choice of a high susceptibility land use and a high infectivity land use. The high susceptibility land use was always ‘broadleaf’, so a parameter combination is defined in terms of its kernel and choice of high infectivity land use. The actual parameter combinations are shown below.

| **parameter** | **Kernel=wide**  **Infe=broadleaf** | **Kernel=wide**  **Infe=agri** | **Kernel=moderate**  **Infe=broadleaf** | **Kernel=moderate**  **Infe=agri** |
| --- | --- | --- | --- | --- |
| $\sigma_{\text{urban}}$ | 0.02 | 0.02 | 0.02 | 0.02 |
| $\sigma_{\text{agri}}$ | 0.02 | 0.02 | 0.02 | 0.02 |
| $\sigma_{\text{broadleaf}}$ | 0.90 | 0.90 | 0.90 | 0.90 |
| $\sigma_{\text{conifer}}$ | 0.02 | 0.02 | 0.02 | 0.02 |
| $\sigma_{\text{semi-n}}$ | 0.02 | 0.02 | 0.02 | 0.02 |
| $\sigma_{\text{wetlands}}$ | 0.02 | 0.02 | 0.02 | 0.02 |
| $\gamma_{\text{urban}}$ | 0.02 | 0.02 | 0.02 | 0.02 |
| $\gamma_{\text{agri}}$ | 0.02 | 0.90 | 0.02 | 0.90 |
| $\gamma_{\text{broadleaf}}$ | 0.90 | 0.02 | 0.90 | 0.02 |
| $\gamma_{\text{conifer}}$ | 0.02 | 0.02 | 0.02 | 0.02 |
| $\gamma_{\text{semi-n}}$ | 0.02 | 0.02 | 0.02 | 0.02 |
| $\gamma_{\text{wetlands}}$ | 0.02 | 0.02 | 0.02 | 0.02 |
| $\lambda$ | 0.34 | 0.34 | 0.67 | 0.67 |
| $\rho$ | 7.50 | 7.50 | 7.50 | 7.50 |
| $\epsilon$ | 0.00 | 0.00 | 0.00 | 0.00 |
