# Supplementary material for: iPAR: A framework for modelling and inferring information about disease spread when the populations at risk are unknown
Source: PLoS Comput Biol. 2025 Jun 16;21(6):e1012622. doi: 10.1371/journal.pcbi.1012622 (PMC12204632; doi:10.1371/journal.pcbi.1012622)
Supplement: S9 Appendix — (DOCX) [file pcbi.1012622.s009.docx]

**Appendix 9: Persistence of infection analysis**

In this section, we take a closer look at the case report data for ASF in wild boar in Estonia. The assumed disease transmission model is a form of spatial SI model, so once a patch becomes infected it remains infected. By looking at the local persistence of ASF infection within each modelled 10km square patch, we can assess how realistic the SI model infection persistence assumption actually is. Within each patch there may be one or more disease case reports. We computed the difference between the first and last disease report time for each patch covering Estonia. A histogram of these differences is shown in Figure A13. We only include patches with reported cases before June 2015 because it is only these patches, infected during the early stages of the outbreak, for which long term persistence of infection can be fully assessed. Infection is usually persistent for at least a year, and often up to four years. The observed persistence of ASF infection in wild boar goes some way to support the SI infection persistence assumption used in the transmission model.


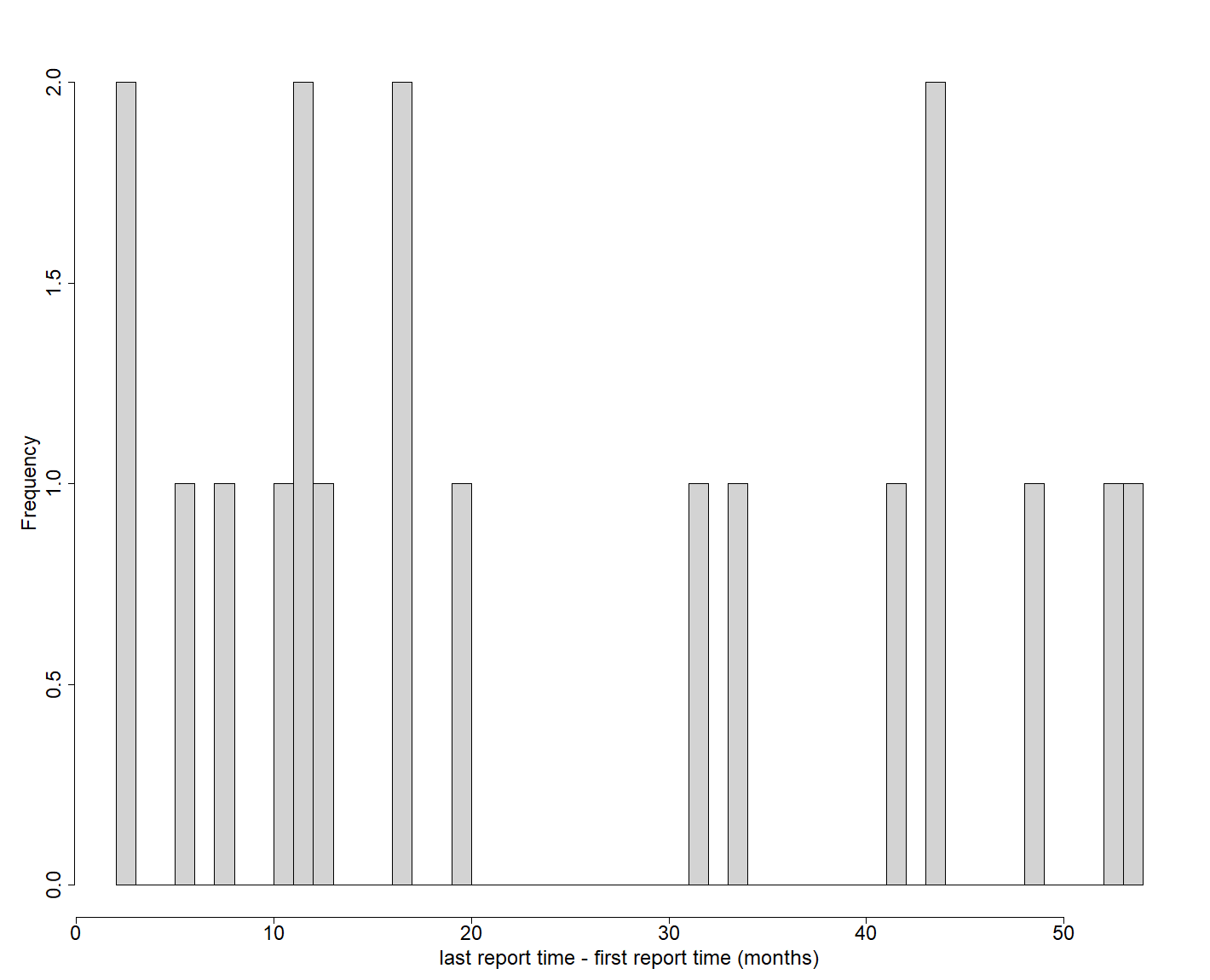


Figure A13. Persistence of infection in 10km square patches covering Estonia. A histogram of the difference between the first disease report time and the last disease report time, as computed for each patch covering Estonia. Patches are excluded if there were no reported cases before June 2015.

One limitation of this analysis is that a relatively small number of patches were infected early on in the outbreak. To gain a broader picture, we also investigated outbreak data from neighbouring countries in the Baltic states – Latvia and Lithuania. Figure A14 shows the same analysis, but conducted over all three countries in the Baltic states. Here, there is even stronger evidence for the long-term persistence of infection in wild boar. Infection usually persists for over 2 years, frequently for 4 or 5 years. This analysis further supports the SI infection persistence assumption used in the transmission model.


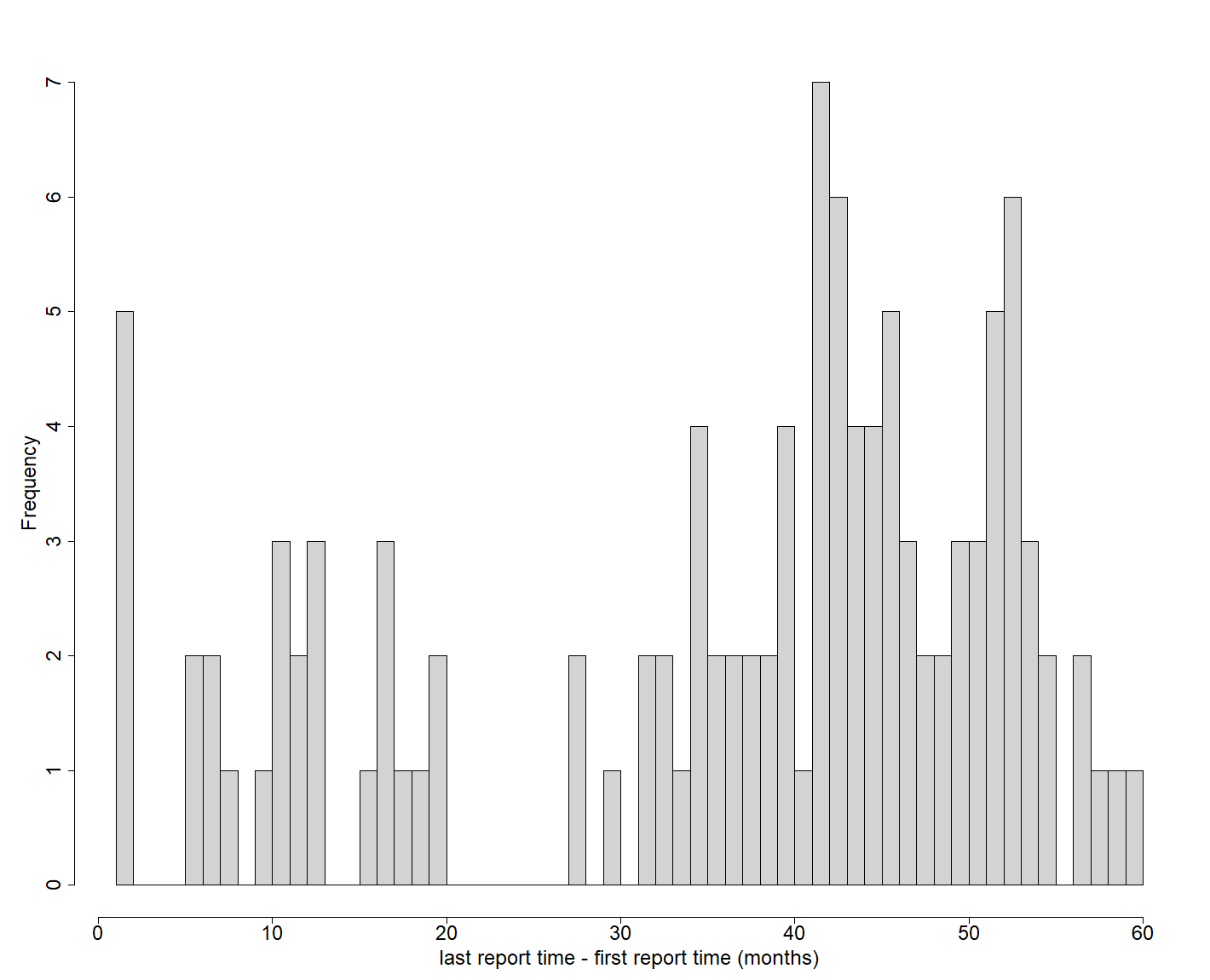


Figure A14. Persistence of infection in 10km square patches covering the Baltic states – Estonia, Latvia and Lithuania. A histogram of the difference between the first disease report time and the last disease report time, as computed for each patch covering the Baltic states. Patches are excluded if there were no reported cases before June 2015.
