# Supplementary material for: iPAR: A framework for modelling and inferring information about disease spread when the populations at risk are unknown
Source: PLoS Comput Biol. 2025 Jun 16;21(6):e1012622. doi: 10.1371/journal.pcbi.1012622 (PMC12204632; doi:10.1371/journal.pcbi.1012622)
Supplement: S10 Appendix — (DOCX) [file pcbi.1012622.s010.docx]

**Appendix 10: Narrow transmission kernel simulations**

This section is motivated by the predictive performance results from the case study, as shown in Table 5 in the main text. The homogeneous and heterogeneous models give very similar predictive performances. Moreover, the predictions seem much better overall than those obtained in Benefits of modelling spatial variation in susceptibility and infectivity in Results – compare Table 5 with Table 1. To help explain these results, we show here the results of simulations similar to those in Benefits of modelling spatial variation in susceptibility and infectivity in Results but with (a) non-kernel parameters close to those estimated from the real data (Table 4), and (b) the kernel parameter set to give various narrow kernels. The precise parameter combinations used in these simulations are shown in the table below. Three distinct parameter combinations were used.

| **parameter** | **Narrow kernel** | **Very narrow kernel** | **Extremely narrow kernel** |
| --- | --- | --- | --- |
| $\sigma_{\text{urban}}$ | 0.01 | 0.01 | 0.01 |
| $\sigma_{\text{agri}}$ | 0.09 | 0.09 | 0.09 |
| $\sigma_{\text{broadleaf}}$ | 0.42 | 0.42 | 0.42 |
| $\sigma_{\text{conifer}}$ | 0.20 | 0.20 | 0.20 |
| $\sigma_{\text{semi-n}}$ | 0.20 | 0.20 | 0.20 |
| $\sigma_{\text{wetlands}}$ | 0.07 | 0.07 | 0.07 |
| $\gamma_{\text{urban}}$ | 0.08 | 0.08 | 0.08 |
| $\gamma_{\text{agri}}$ | 0.82 | 0.82 | 0.82 |
| $\gamma_{\text{broadleaf}}$ | 0.03 | 0.03 | 0.03 |
| $\gamma_{\text{conifer}}$ | 0.02 | 0.02 | 0.02 |
| $\gamma_{\text{semi-n}}$ | 0.03 | 0.03 | 0.03 |
| $\gamma_{\text{wetlands}}$ | 0.03 | 0.03 | 0.03 |
| $\lambda$ | 1.45 | 3.00 | 5.00 |
| $\rho$ | 4.54 | 4.54 | 4.54 |
| $1000\epsilon$ | 7.65 | 7.65 | 7.65 |

The predictive performance obtained in these simulations is shown in the table below. The results obtained are similar to those obtained in Table 5 in the case study i.e. very high discriminatory power, and close agreement between the heterogeneous and homogeneous fitted models.

|  |  | **Discrimination metrics** | | ${N_{est}}/{N_{truth}}$ | |
| --- | --- | --- | --- | --- | --- |
| **Kernel** | **Model** | **mean AUC** | **mean TPR @ 5% FPR** | **reliability (%)** | **mean bias** |
| Narrow | homog | 0.77 | 0.22 | 95 | 0.02 |
|  | heter | 0.77 | 0.23 | 100 | 0.01 |
| Very narrow | homog | 0.84 | 0.37 | 95 | 0.04 |
|  | heter | 0.85 | 0.39 | 100 | 0.02 |
| Extremely narrow | homog | 0.86 | 0.36 | 95 | 0.04 |
|  | heter | 0.86 | 0.38 | 100 | 0.02 |
