# Supplementary material for: iPAR: A framework for modelling and inferring information about disease spread when the populations at risk are unknown
Source: PLoS Comput Biol. 2025 Jun 16;21(6):e1012622. doi: 10.1371/journal.pcbi.1012622 (PMC12204632; doi:10.1371/journal.pcbi.1012622)
Supplement: S11 Appendix — (DOCX) [file pcbi.1012622.s011.docx]

**Appendix 11: spread of ASF in Estonia at different spatial scales**

In the case study in the main manuscript, we model the spread of ASF in wild boar in Estonia at the 10km resolution. That is, the patches in the model are 10km squares. Here, we briefly explore modelling the same set of outbreak data but at a different, higher spatial resolution of 5km. It is not immediately obvious how the parameter estimates obtained at different resolutions might be related, especially the susceptibility and infectivity estimates for the land use classes in the land use data. The land use covariates, obtained by aggregating over each patch, could be very different at different spatial resolutions. For example, the landscape might seem homogeneous at a coarse resolution – in the sense that each patch has the same land use composition – but at a finer resolution there may be a much higher degree of between-patch variation in land use composition.

**5km spatial resolution: spatially aggregated outbreak data**

First, we explore the spatially aggregated outbreak data at different points in time. Figures A10, A11, A12 show how the infection spreads across Estonia, at the 5km resolution. These figures are analogous to Figure 6 in the main manuscript, but at this higher spatial resolution. There is clear visual evidence of distance-dependence in the spread of disease.


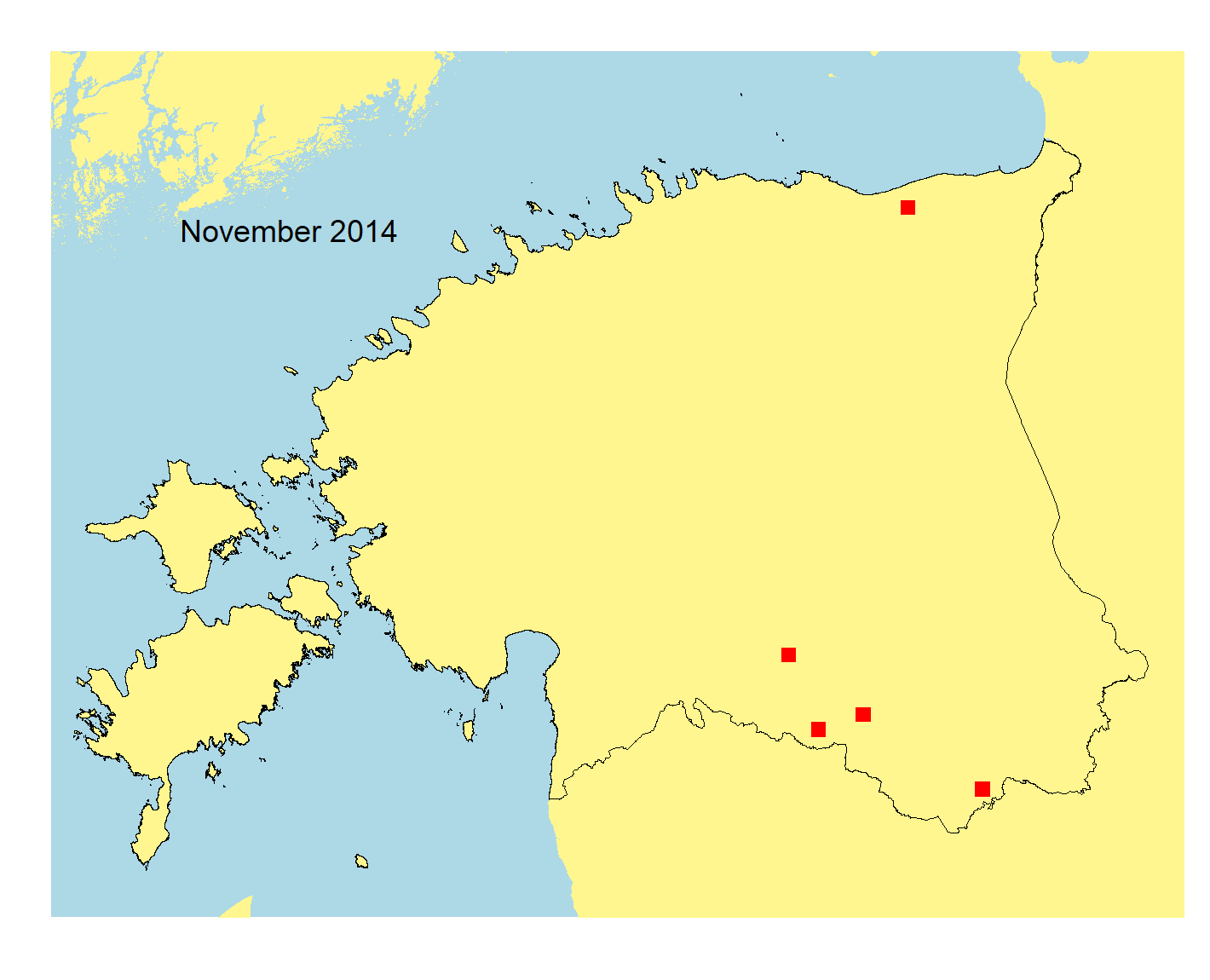


Figure A10. **Case reports**: Spatially aggregated (5km resolution) African Swine Fever wild boar cases reported in Estonia. Snapshot as at November 2014. Map base layer taken from https://gadm.org/maps.html.

**
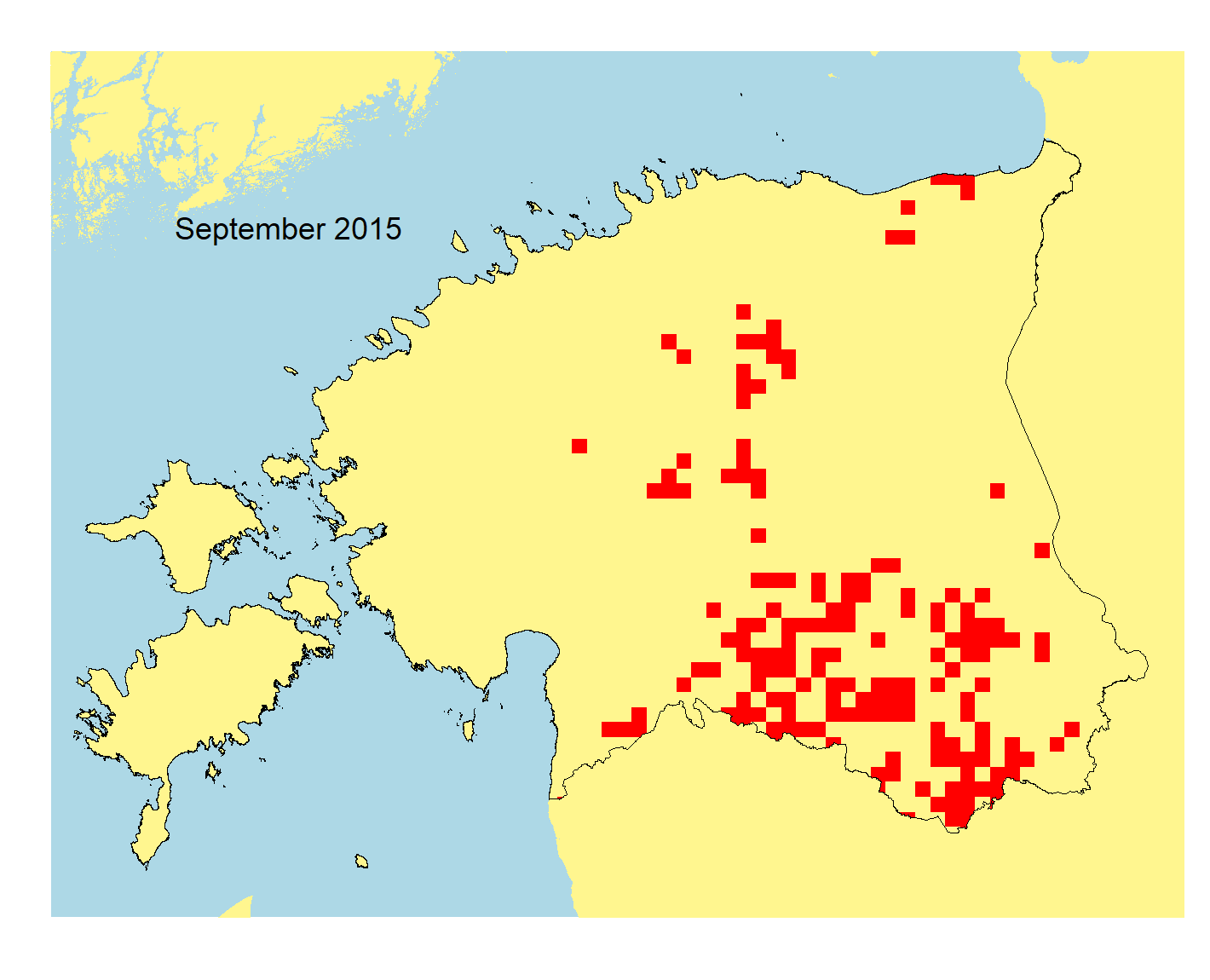
**

Figure A11. **Case reports**: Spatially aggregated (5km resolution) African Swine Fever wild boar cases reported in Estonia. Snapshot as at September 2015. Map base layer taken from https://gadm.org/maps.html.

**
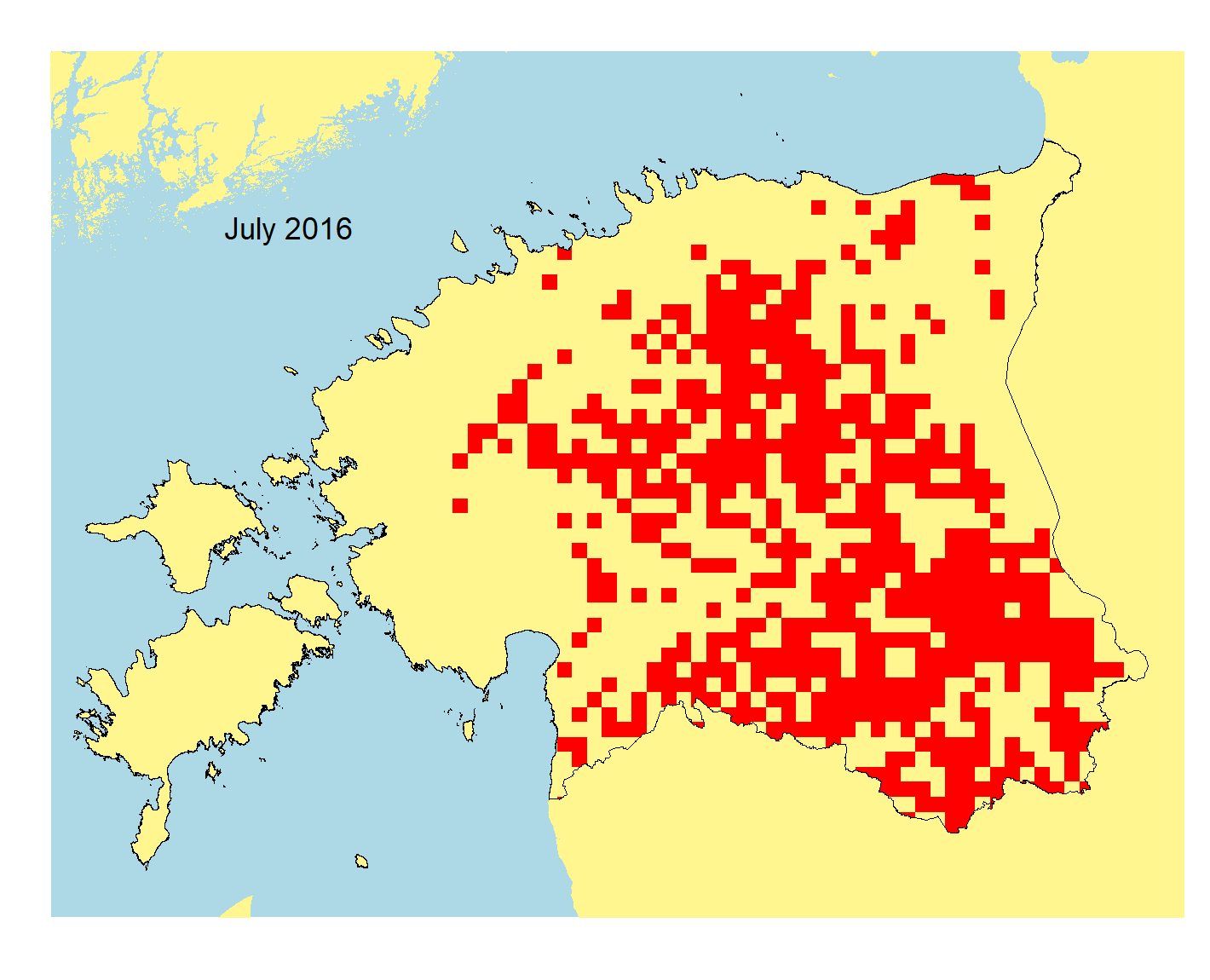
**

Figure A12. **Case reports**: Spatially aggregated (5km resolution) African Swine Fever wild boar cases reported in Estonia. Snapshot as at July 2016. Map base layer taken from https://gadm.org/maps.html.

**5km spatial resolution: model parameter estimates and interpretation**

We fitted the constant-in-time model to the same set of outbreak data as used in the case study in the main text, but at the 5km spatial resolution. The structure of the model is the same as that used in the case study in the main text, except for the higher spatial resolution. Parameter estimates from the fitted model are shown below.

|  | **Estonia constant in time** | | |
| --- | --- | --- | --- |
| **parameter** | **lower** | **median** | **upper** |
| $\sigma_{\text{urban}}$ | 0.00 | **0.01** | 0.06 |
| $\sigma_{\text{agri}}$ | 0.19 | **0.26** | 0.34 |
| $\sigma_{\text{broadleaf}}$ | 0.20 | **0.28** | 0.37 |
| $\sigma_{\text{conifer}}$ | 0.13 | **0.21** | 0.29 |
| $\sigma_{\text{semi-n}}$ | 0.05 | **0.17** | 0.29 |
| $\sigma_{\text{wetlands}}$ | 0.01 | **0.05** | 0.10 |
| $\gamma_{\text{urban}}$ | 0.00 | **0.08** | 0.34 |
| $\gamma_{\text{agri}}$ | 0.28 | **0.50** | 0.74 |
| $\gamma_{\text{broadleaf}}$ | 0.01 | **0.13** | 0.34 |
| $\gamma_{\text{conifer}}$ | 0.00 | **0.06** | 0.20 |
| $\gamma_{\text{semi-n}}$ | 0.00 | **0.05** | 0.23 |
| $\gamma_{\text{wetlands}}$ | 0.00 | **0.09** | 0.31 |
| $\lambda$ | 1.60 | **1.87** | 2.30 |
| $\rho$ | 0.57 | **0.76** | 1.09 |
| $1000\varepsilon$ | 27.56 | **33.32** | 39.64 |

When interpreting the above parameter estimates, it is worth bearing in mind that the estimates may not necessarily match those obtained at the 10km resolution. The pattern of disease spread may appear quite different at different spatial resolutions. Nevertheless, the susceptibility and infectivity estimates are broadly similar to those at the 10km resolution, as can be seen by comparing the table above with Table 4 in the main text. Broadleaf woodland remains the land use with the highest susceptibility, while agricultural land remains the land use with the highest infectivity.

Regarding estimates of the transmission kernel parameter $\lambda$*,* it is worth remembering that distance is defined in terms of the patch dimensions, so when the patch size is reduced we also change the scale on which distance is measured. The power law transmission kernel is scale invariant, so we might perhaps expect the estimates of $\lambda$ to be similar at different spatial scales. On the other hand, the transmission is unlikely to exactly follow a power law kernel. What we actually find is that $\lambda$ is estimated to be a little higher at 5km resolution, suggesting something a little closer to ‘nearest neighbour’ transmission between patches. The overall rate $\rho$ is also lower, while the background transmission rate $\varepsilon$ is higher. In summary, background transmission seems relatively more important when the data are viewed at the 5km resolution.
