# Supplementary material for: iPAR: A framework for modelling and inferring information about disease spread when the populations at risk are unknown
Source: PLoS Comput Biol. 2025 Jun 16;21(6):e1012622. doi: 10.1371/journal.pcbi.1012622 (PMC12204632; doi:10.1371/journal.pcbi.1012622)
Supplement: Attachment — Submitted filename: IPAR_framework_review.docx [file pcbi.1012622.s012.docx]

**Review of manuscript :**

**« iPAR: A framework for modelling and inferring information about**

**disease spread when the populations at risk are unknown »**

**Stephen Catterall, Thibaud Porphyre and Glenn Marion**

*Abstract*

The limits of the current approaches are a bit vague. I understand you’re constrained in words but the gap you’re trying to fill could be more precise for the abstract to be more appealing.

*Introduction*

RAS ?

*2. Ipar framework*

*2.1 The generic model*

I think your appendices should be cited in the main text in the order they appear in your ESM. Currently Appendix 2 is cited first.

What does « I » stand for in your kernel ?

I don’t understand why can patch indices i and j be squared in your normalization constant, overall I must be missing something for how this constant works, despite your explanation. As you don’t cite any reference for this truncated power law kernel, I’m assuming you designed it, so you could expand you Appendix 2 to be more didactical and maybe add graphical explanations of how this kernel works.

You don’t provide line numbers so I can’t precisely tell you where to look, but I’m guessing you made a typo when referring to susceptibility and infectivity of patch i as S_i_ and I_i_ at the beginning of a paragraph, because you use s_i_ and t_i_ everywhere else.

When you explain your rationale for how absolute and relative covariates influence susceptibility and infectivity : I think there’s a bit of restructuring to be done. The change in paragraph doesn’t seem relevant where it is currently placed, as the properties of sigma_l and sigma_k end up separated.

“However, it is important to note that negative relationships are also possible for the land use covariates, even though the sigma_l are non-negative, due to the constraint that ∑ℎ_𝑖,𝑙_ = 1.” Shouldn’t it be sigma_l in the sum?

“For example, the covariate ℎ_𝑖,𝑙_ with the smallest associated sigma_l will be negatively related to susceptibility.” I don’t understand this sentence : is the smallest sigma_l necessarily a negative association? Can we have only positive associations? Can we have more than one negative association (not only the smallest)? This sentence doesn’t add clarity to the statement saying that negative associations can be incorporated even with only positive parameters.

*2.2 Bayesian inference and prediction based on disease case reports*

*2.2.1 Parameter and latent variable estimation*

To do: check appendix 1,3,4

I don’t have the skills to properly evaluate the paragraph with the likelihood formula.

*2.2.2 Model selection*

I would move the first sentence towards the end of the paragraph, as this is not the main idea developed in the paragraph but rather an explanation of why you don’t dive further into the subject.

*2.2.3 Uncertainty in model predictions*
