# Supplementary material for: iPAR: A framework for modelling and inferring information about disease spread when the populations at risk are unknown
Source: PLoS Comput Biol. 2025 Jun 16;21(6):e1012622. doi: 10.1371/journal.pcbi.1012622 (PMC12204632; doi:10.1371/journal.pcbi.1012622)
Supplement: Attachment — Submitted filename: Response to reviewers.docx [file pcbi.1012622.s014.docx]

Biomathematics and Statistics Scotland

JCMB

The King’s Buildings

Peter Guthrie Tait Road

Edinburgh EH9 3FD

14^th^ April 2025

Dear Editors,

**Revised version of PCOMPBIOL-D-24-01946**

Thank you very much for considering our manuscript “iPAR: A framework for modelling and inferring information about disease spread when the populations at risk are unknown” for publication in PLOS Computational Biology. We have addressed the reviewer’s comments in the revised version of the manuscript, attached herewith, and believe that these changes have significantly strengthened the paper.

In this letter we list each comment made by the referee/editor, together with our response. **The line numbers quoted refer to the attached revised manuscript with tracked changes, unless stated otherwise**. *Reviewer/editor comments are in italics*. Quotes are taken from the revised manuscript.

***Additional Editor Comments:***

*The reviewers appreciated the importance of the problem you are addressing, and innovative nature of your proposed solution. They also provided detailed suggestions for improving the clarity and utility of your manuscript by including additional discussion of:*

*factors such as data availability, selection of covariates, computational cost, that may be relevant to use of the proposed methods by other researchers.*

**Response**: We have made many changes to the manuscript with the aim of providing additional guidance that might help readers how to use the proposed framework in practice. Guidance on covariate selection has been added in the Introduction at Page 3, Lines 37-40 and in Methods at Page 4, Lines 19-20 and in the Discussion at Page 22, Lines 20-25. Computational cost is discussed at Page 10, Lines 12-21.

*the applicability of the proposed methods to populations and diseases with different characteristics (eg, human populations, recovery/immunity)*

**Response**: We have added text discussing the potential applications of the framework to other populations (Page 3, lines 37-40, Page 4, Lines 11-13). We have also discussed diseases with different characteristics (Page 4, Lines 29-36, Page 24, Lines 12-20). We emphasise that the framework could be applied to disease in humans, animals and plants – but for humans there may be less of an issue with uncertainty over the population itself, but uncertainty over the population *at risk* may nonetheless be considerable. We also emphasise that the disease compartments used to model disease spread at the patch scale may be different from the compartments used to model disease spread at the individual level.

*how your approach compares to existing modelling methods.*

**Response**: We believe that we have given a good overview of existing approaches in the Introduction. Reviewer 3 asked about the potential for comparison between iPAR and other approaches – we refer to our response to Reviewer 3 on Page 10 of this letter. In summary, a full simulation study would be difficult and beyond the scope of our paper which we did not want to add to given the comments of Reviewers 2 & 4 on the length of the current manuscript. However, we’ve added additional text to discuss comparison between methods in more detail. We also highlight that ‘Benefits of modelling spatial variation in susceptibility and infectivity’ in Results is essentially a comparison, via simulations, of the iPAR approach to a simpler model which ignores spatial variation in the population at risk, whilst still accounting for depletion of susceptibles.

*While the provision of code was appreciated, clearer documentation would make your methods more readily usable by others.*

**Response**: We have added a pdf file to the github repository at <https://github.com/spcatterall/iPAR> which contains detailed information about how to run the code provided. We also include example input files for both the simulation code and the inference code in order to help users run the code.

***Reviewer #1****: Abstract*

*The limits of the current approaches are a bit vague. I understand you’re constrained in words but the gap you’re trying to fill could be more precise for the abstract to be more appealing.*

**Response**: We have made the text of the abstract more concise and explicit (Page 1, lines 10-15), to emphasise that typical modelling approaches require spatial data on the disease host, and this requirement defines the gap that we are trying to fill – or, putting it another way, the condition that we are trying to relax.

*Introduction*

*RAS ?*

**Response**: We didn’t understand what this comment meant.

*2. Ipar framework*

*2.1 The generic model*

*I think your appendices should be cited in the main text in the order they appear in your ESM. Currently Appendix 2 is cited first.*

**Response**: Thank you for spotting this error. We have corrected the numbering of the Appendices in the revised manuscript.

*What does « I » stand for in your kernel ?*

**Response**: A definition has been added to the text at Page 5, lines 9-10: “I is an indicator function (equal to 1 if its argument is a true statement, 0 otherwise)”.

*I don’t understand why can patch indices i and j be squared in your normalization constant, overall, I must be missing something for how this constant works, despite your explanation. As you don’t cite any reference for this truncated power law kernel, I’m assuming you designed it, so you could expand you Appendix 2 to be more didactical and maybe add graphical explanations of how this kernel works.*

**Response**: Apologies for any confusion, there was a slight inconsistency in notation between the main text and the appendix. We have modified the notation in the appendix, which now appears as Appendix 1, and have added additional explanation there. We have also added a reference in the main text to an older publication (Cook et al. 2007) which used the same approach (Page 5, line 11).

*You don’t provide line numbers so I can’t precisely tell you where to look, but I’m guessing you made a typo when referring to susceptibility and infectivity of patch i as Si and Ii at the beginning of a paragraph, because you use si and ti everywhere else.*

**Response**: Apologies for the absence of line numbers in the initial submission. We have corrected the typo (Page 5, line 25).

*When you explain your rationale for how absolute and relative covariates influence susceptibility and infectivity : I think there’s a bit of restructuring to be done. The change in paragraph doesn’t seem relevant where it is currently placed, as the properties of sigma_l and sigma_k end up separated.*

**Response**: We have restructured the last two paragraphs of this section (Page 5, line 25 onwards). The penultimate paragraph introduces the models for both infectivity and susceptibility, while the last paragraph highlights some properties of the assumed model.

*“However, it is important to note that negative relationships are also possible for the land use covariates, even though the sigma_l are non-negative, due to the constraint that ∑ℎ𝑖,𝑙 = 1.” Shouldn’t it be sigma_l in the sum?*

**Response**: We have checked this section and the quoted sentence is in fact correct. However, we can see that the presentation was not very clear so we have added further explanation which we hope will be helpful (Page 6, lines 12-15):

“…due to the constraint that $\sum_{l=1}^{L} h_{i,l}=1$. For example, if the smallest component of $\sigma$ is $\sigma_{q}$ then the covariate $h_{i,q}$ will be negatively related to susceptibility: if $h_{i,q}$ increases then $\sum_{l\neq q} h_{i,l}$ will correspondingly decrease, and the overall effect is that $\sum_{l=1}^{L} h_{i,l}\sigma_{l}=h_{i,q}\sigma_{q}+\sum_{l\neq q} h_{i,l}\sigma_{l}$ will decrease.”

*“For example, the covariate ℎ𝑖,𝑙 with the smallest associated sigma_l will be negatively related to susceptibility.” I don’t understand this sentence : is the smallest sigma_l necessarily a negative association? Can we have only positive associations? Can we have more than one negative association (not only the smallest)? This sentence doesn’t add clarity to the statement saying that negative associations can be incorporated even with only positive parameters.*

**Response**: We have rewritten this section to clarify the intended meaning and we refer to our response to your comment directly above this one. The covariate associated with the smallest component always has a negative association, while the covariate associated with the largest component always has a positive association. For other components the nature of the association will depend on the precise values of the quantities involved.

*2.2 Bayesian inference and prediction based on disease case reports*

*2.2.1 Parameter and latent variable estimation*

*To do: check appendix 1,3,4*

*I don’t have the skills to properly evaluate the paragraph with the likelihood formula.*

**Response**: We have checked the expression for the likelihood (now moved to Appendix 3) as well as the associated appendices. We don’t believe that any corrections are needed.

*2.2.2 Model selection*

*I would move the first sentence towards the end of the paragraph, as this is not the main idea developed in the paragraph but rather an explanation of why you don’t dive further into the subject.*

**Response**: We have changed the text as suggested (Page 8, lines 1-2).

*2.2.3 Uncertainty in model predictions*

***Reviewer #2****: The manuscript introduces the iPAR (inference for populations at risk) framework, a novel modeling and inference approach for spatial infectious disease dynamics when the population at risk is uncertain or poorly quantified. This is a non-trivial and important problem in epidemiology and population health, where precise host distribution data are frequently unavailable. The proposed approach is both conceptually interesting and methodologically innovative. It attempts to extract critical epidemiological information—such as susceptibility, infectivity, and rate of spread—from case-only data, supplemented by spatial covariates. The manuscript includes a theoretical and methodological development, as well as simulation-based evaluations and a real-world case study on African Swine Fever (ASF) in Estonian wild boar populations.

\# Strengths:

The introduction of the iPAR framework addresses a critical gap in spatial disease modelling, especially when populations at risk are poorly quantified. This framework is highly relevant for wildlife outbreaks and under-documented livestock settings.

The step-by-step development of the model, including Bayesian inference, patch-based structure, and the use of covariates, is thoroughly detailed.

The authors show that the method can incorporate spatial covariates—land use, climate factors, or other relevant environmental proxies—to estimate susceptibility and infectivity surfaces. The framework is not restricted to a specific host-pathogen system and can be extended, at least in principle, to more complicated compartmental structures or multiple host species.*

**Response**: We thank the reviewer for recognizing the value of the iPAR framework.

*# Potential limitations and suggestions (major comments):

While the paper acknowledges the difficulty of recovering infectivity parameters compared to susceptibility, it may be beneficial to discuss conditions under which identifiability issues arise more explicitly. For instance, what levels of data availability (in terms of number of cases, spatial resolution, and outbreak duration) are needed for robust inference of both susceptibility and infectivity?*

**Response**: This is a very reasonable question. We have added more text to the discussion to provide some guidance. The number of cases required will depend on the model structure and the covariates, so it’s hard to be too prescriptive. We’ve highlighted some other works that touch on this issue, and have also given a rough rule of thumb based on our own experience with the iPAR approach (Page 23, lines 1-11):

“It is challenging to precisely define the level of outbreak data availability needed to robustly estimate model parameters. Simple spatial spread models with no variation between infectious units can be fitted even when there are less than 10 reported disease cases (Gamado, Marion, and Porphyre 2017). Larger numbers of cases may be required for precise estimates when fitting more complex models. Analytic expressions for the precision of parameter estimates have been derived for non-spatial models that allow for variation in susceptibility and infectivity (C. Pooley et al. 2022). To successfully fit the iPAR model, the required number of cases will depend on the model structure and choice of spatial covariates. For the scenarios considered in this paper, we found that – as a very rough rule of thumb - between 10 and 100 cases are required to obtain robust parameter estimates. The duration of time over which the cases used to fit the model are collected is of lesser importance than the number of cases.”

*The authors note that the method can use a wide range of covariates. However, more guidance on how to select appropriate covariates, how to handle spatio-temporal variation in these covariates, and how to choose the patch size would strengthen the applicability. While patch-based models are common, best practices or decision criteria for resolution selection would be useful, especially in more heterogeneous landscapes or when data resolution differs between outbreak reports and covariates.*

**Response**: We have added more guidance on selection of covariates e.g. Page 3, lines 37-40:

“The precise choice of covariates will depend on the specific application at hand and the availability of spatially explicit covariates. In the case of a human disease, relevant covariates could include local human population density and key indices e.g. measuring social deprivation and air pollution.”

Also Page 4, lines 19-20:

“In practice, we choose covariates that are likely to explain at least some of the variation in host density across the region”

Also Page 22, lines 22-25:

“If spatial population density estimates are available then they could be used as covariates in the model, possibly with a strong prior attached to the associated effect if we believe that the estimates are particularly informative.”

We have added more guidance on choice of patch size e.g. see Page 4, lines 25-28:

“The chosen size of the patches depends on a number of factors, including the spatial resolution of the covariates and the disease case data. If the covariates have a different spatial resolution to the case data then one or both data sets may be aggregated or de-aggregated to the chosen resolution. Patch size should be small enough to capture the apparent pattern of disease spread across the region.”

As mentioned in the methods, time varying covariates could in principle be handled within the iPAR framework but are outside the scope of the current implementation. We also highlight the paragraph in the discussion (Page 23, lines 33-42) which covers robustness of model outputs to the choice of patch size. Spatial heterogeneity of the transmission process is naturally handled within the iPAR framework via the use of spatial covariates. The choice of covariates is highly problem dependent, but iPAR provides a framework within which researchers can test the suitability of different sets of covariates, for example by comparing the resulting fitted models in terms of key predictive performance metrics and DIC values.

*The current work focuses on an SI-type model with persistence. Many real-world diseases involve recovery, waning immunity, or multiple host types. A clearer roadmap on how to incorporate these complexities within the iPAR framework would strengthen its applicability. While mentioned as a future direction, more concrete guidance or at least a detailed conceptual outline would improve the paper’s practical utility.
In addition, clearly state the implications of using the SI model, especially for diseases like ASF that have latent phase, or other that would have a recovery phase.
Moreover, since inference is based on a likelihood-based approach, the use of a more complex model seems likely to severely hamper the use of the proposed framework. It is essential at the start of the article to clearly define the conditions of application of the framework.*

**Response**: This is a very reasonable comment/question. The current implementation of iPAR, as expressed in the code provided, covers the scenarios presented in the paper, but also allows for more general sets of covariates. We have added some text covering generalizations to more complex model structures beyond the scope of the current implementation (Page 24, lines 12-20). Certainly, if the transmission model was more complex then an alternative approach to inference could be required. We have also now mentioned the latent period of ASF in the case study (Page 17, lines 41-44), and how it is short relative to the timescale of the apparent spread. We should also emphasise that the characteristics of the disease at the individual host level, in terms of disease compartments, is not necessarily the same as the characteristics at the patch level. For example, in the case of ASF in wild boar in Estonia, we have established that we can use an SI model to represent the data at the patch scale that we describe in the paper (and several other patch-scales that we explored in our analysis) despite the fact that at an individual-host level ASF does not follow an SI dynamic. This point has been emphasised in the text (Page 4, lines 30-33).

*The methods may be computationally intensive for large-scale problems with many patches and long observation periods. Although the paper mentions computational tractability as a motivation for patch aggregation, a discussion of computational scaling or potential performance optimizations (e.g., parallelization, approximation methods) would be valuable. No indication is given of the language used or the computing resources consumed (time, memory), which would give a better idea of the framework's performance and potential for improvement.*

**Response**: We have provided more information on the code (Page 7, lines 34-35) and its running time (Page 10, lines 12-21). There is also further information in the newly added documentation in the github repository. We emphasise that, for the problems that we describe, fitting the model isn't hugely computationally intensive. For example, for the case study, from a purely computational point of view the model could be updated daily using modest computational resources such as a few hours on a basic laptop. This suggests that the methodology presented is practically applicable for real-world applications.

*In "2.2.3 Uncertainty in model predictions" : "...initial conditions typically set to the infection status of the patches at the final time point of the
data used to fit the model ( = ). ...". In this case, it's assumed that the system is perfectly observed, but it's highly unlikely, and often impossible, to observe this kind of system exhaustively. Why not use as initial conditions the state of the system at t=T obtained in the simulations carried out? If the data do not contain an observation of all the patches, then it is very likely that the assumption made about the initial conditions tends to minimize the number of infected patches.*

**Response**: In line with many other authors (see additional references added at Page 6, lines 37-39) we do make the common assumption of perfect observation, and relaxing this assumption, for example along the lines suggested, is outside the scope of the current manuscript. However, we do comment that in the iPAR framework the impact of this assumption is significantly mitigated by both the patch-scale and the interval censoring e.g. in the case study we assume that if infection occurs within a hectad (10x10km grid cell) it will be detected within a year under conditions of high vigilance for ASF. The chance that such an infection is missed will rapidly decrease as the extent to which the patch is infected increases - think of multiple binomial trials at the sub-patch scale conducted by surveillance annually. Thus, patches missed by such surveillance will be highly likely to suffer low levels of infection and play an insignificant role in transmission and therefore not seriously impact the ability of the iPAR framework to capture the dynamics of between patch transmission. This expectation is borne out by the predictive performance of iPAR in the case study.

*In "2.2.4 Testing the iPAR modelling approach" : This type of approach (the use of synthetic/simulated data) also has its limitations and drawbacks, which should be mentioned and discussed (choice of synthetic trajectory, etc.).*

**Response**: This is a good point. We’ve added text on the limitations of using simulated data (Page 8, lines 43-44). This is one reason why we conducted a case study based on a real world application and real world data.

*In "3.1 Estimation and prediction for an illustrative simulated outbreak" and "3.2 Estimation of key epidemiological parameters" : In these sections, no mention is made of how the simulated trajectory used as an observation was chosen, even though this can have a significant impact on the results. It should be described and discussed.*

**Response**: In ‘Estimation and prediction for an illustrative simulated outbreak’ in Results, the parameters are taken to be similar to those estimated in Case study (Page 10, lines 24-26). In ’Estimation of key epidemiological parameters’ in Results, the parameters used represent perturbations from the baseline parameterization in the previous section. In all cases, simulated trajectories were chosen at random conditional on the model parameters – a sentence has been added to record this (Page 10, line 19).

*The approach infers “effective” susceptibility and infectivity surfaces that combine unknown population density and other factors. This layered interpretation may cause confusion for end-users. More explicit statements about the interpretative limits of these inferred surfaces, and how to use them alongside or in the absence of independent population density estimates—would help ensure that the results are not misinterpreted as direct host density maps.*

**Response**: We have tried to be clear that the surfaces are not population density estimates. This is mentioned in the Introduction (Page 3, lines 17-19), and some text has also been added to the first paragraph of the Discussion (Page 22, lines 20-25) – where we have also mentioned how population density estimates might be incorporated into an analysis.

*While the paper’s accompanying code is appreciated, it appears incomplete. It lacks clear guidance on compilation and execution (notably due to the C code), and there are no provided examples or instructions for the necessary input files. More comprehensive documentation is mandatory.*

**Response**: Apologies for the lack of documentation in the github repository. We have added a document describing how to run the code, compile it, etc. We have also included some data files that can be used as input files for the code, with full explanations of what the inputs represent. Finally, we explain the outputs of the simulation/inference code in a way that allows them to be used for further processing e.g. to create risk maps.

*# Minor comments:

- Define "external transmission" explicitly to ensure clarity for readers less familiar with disease modelling.*

**Response**: Thank you for pointing this out. External transmission was not explicitly mentioned when we first introduced the expression for the force of infection (equation 1). We have now corrected this (Page 5, line 3).

*Figures are informative, but their descriptions could be more concise. Ensure that captions focus on the key message of each figure (e.g., “parameter recovery demonstrates…”). For few figures, the addition of a legend would make reading easier.*

**Response**: Where necessary we have edited figure captions to try to ensure that the key message is immediately apparent.

*Part of the penultimate paragraph of the introduction (first half) seems to belong to the second part, which focuses on method.*

**Response**: We couldn’t see any particular issue with the penultimate paragraph so haven’t changed it.

*Tables : In the definition of scenarios, the "Susceptibility" variable always seems to be the same, and therefore not very useful to have it in the tables.*

**Response**: This is a valid point. On the other hand, we wanted to include Susceptibility in order to emphasise that it **is** always the same, so we haven’t removed this column.

*# Summary:

It's an interesting paper with valuable insights, but it is quite lengthy and dense, which can make it challenging for the reader to maintain a clear grasp of the material, and make it easy to occasionally lose track of the main thread, but well worth reading.
This manuscript makes a valuable contribution to spatial disease modelling, particularly for scenarios where host population data are limited or unavailable.
Addressing the suggested clarifications on model limitations, assumptions, guidance, and extended discussion on model complexity would further enhance the manuscript.*

**Response**: Thanks for your comments. We have tried to add further clarifications as suggested. Unfortunately, this has added a little to the length of the manuscript. We believe that the manuscript is as short as possible without deleting some key material.

***Reviewer #3****: iPAR: A framework for modeling and inferring information about disease spread when
the populations at risk are unknown

comments:
The authors developed the iPAR framework which enables modeling and estimating of the spatial disease dynamics when the populations at risk are unknown. The authors presented rigorous simulation analysis with different spatiotemporal scenarios and real-world data analysis results. It is very interesting and relevant work since there are many situations we need to make strong assumptions about susceptible populations. This work can contribute to the infectious disease literature providing the modeling framework with a more accurate and flexible approach when we have the incomplete population distribution. However I have a few questions regarding the iPAR framework described in the manuscript.*

**Response**: Thanks for your comments.

 *I had the impression that the iPAR framework targets the infectious disease of animals, even though the manuscript did not specifically mention it. Can it be applied to human infectious disease transmission modeling? Is there any appropriate scenario of human infectious disease that the iPAR framework can be well suited? If it can be, I think it is necessary to show i) how iPAR model terms are changed or similarly used in human infectious disease transmission modeling, ii) which covariates can be applied to estimate surface infectivity and susceptibility, in case of human transmission, iii) how it can be used to model and predict infectious human disease transmission in simulation analysis. Currently, the model covariates ( e.g. Land use), simulation scenarios and real-world applications all focus on animal infectious disease modeling. If not, the manuscript needs to specifically mention that this paper mainly targets animal infectious disease transmission to help better understanding of audiences.*

**Response**: The framework is generally applicable to disease spread in humans, plants and animals including crops and livestock but it would be necessary to select appropriate covariates in each case. We have provided a case study based on wildlife disease. The framework could be extended to human case only data, but for humans there may well be better data on host distribution e.g. population density estimates that could be utilized within the iPAR framework. In addition, the risk factors that one might consider for human diseases would likely be different from those for plants/ animals and different again for farmed livestock and crops. For human diseases one might consider indices of deprivation within the human population as a risk factor. However, some environmental covariates could be relevant to specific human infectious diseases for example long-term air pollution has been shown to be important for susceptibility to some respiratory diseases. We have added this text at Page 3, lines 37-40:

“The precise choice of covariates will depend on the specific application at hand and the availability of spatially explicit covariates. In the case of a human disease, relevant covariates could include local human population density and key indices e.g. measuring social deprivation and air pollution.”

We also added at Page 4, lines 11-13:

“In principle, any disease could be modelled, whether of animals, plants or humans. However, the case study presented focusses on animal disease.”

*2. External Validation: This is a novel framework to model infectious disease transmission when the population at risk is unknown. As the authors wrote in section 1, there exist previous modeling methods based on transmission trees, contact-distribution models, and approaches based on hypothesized population distributions. What will be the benefits of using the iPAR framework compared to existing approaches? How will model performance be different ( e.g. DIC, TPR and FPR) among different approaches? In which simulation scenario does the iPAR perform better than other existing approaches? The authors can show it with different simulation analyses.*

**Response**: Such a simulation study would be outside the scope of the current manuscript, given that the manuscript is already very long and it would be time-consuming to actually implement the alternative approaches published by other authors. However, we also highlight that these alternative approaches address different aspects of the problem – so that comparison between them is not straightforward e.g. not all of them offer predictions of future spread. We’ve added text in the discussion to emphasize this point (Page 22, lines 26-34), and also to highlight that, in our view, the iPAR framework is the most comprehensive solution, to-date, that addresses the problem of unknown populations at risk. Furthermore, we highlight that ‘Benefits of modelling spatial variation in susceptibility and infectivity’ in Results is essentially a comparison, via simulations, of the iPAR approach to a simpler model which ignores spatial variation in the population at risk, whilst still accounting for depletion of susceptibles (something which other competing approaches such as Lau et al. (2017) and MacCalman et al. (2017) fail to do.)

*3. The iPAR framework uses case reports of the region, but it only estimates and predicts whether the patch itself is infected or not. Is the small/ large number of cases in each patch associated with the performance of the iPAR framework? Or is it only a binary decision that is important here (patch is infected at time t, or not)? It would be a relevant question whether the performance of the iPAR framework will be the same for the regions (multiple patches combined) with only a small number of cases and with a large number of cases, similar to the small area estimation problem in aggregated spatial entities. Will the performance metrics or the width of 95% credible intervals of estimators be the same for these areas with small and large cases? Simulation or real-world data analysis can show this difference.*

**Response**: Much like dividing the population into compartments such as susceptible, infectious and recovered, dividing the population into patches also has a consequence in terms of how one interprets the results from a fitted model. Nonetheless, similar to the division of individuals into disease states our results show that we can gain valuable information by fitting models at the patch scale considered. Interpreting the results provides not only quantification of the impact of disease control but also estimates rates of disease spread and susceptibility and infectivity at the landscape scale with a resolution defined by the patches. Together this information enables quantitative risk assessments that can be used to inform disease control and surveillance. As it stands, the iPAR model framework doesn’t distinguish between patches with large numbers of cases and patches with small numbers of cases. Once the first case has been reported, all following cases are irrelevant from the perspective of the model. What we can say is that changing the patch size from 10km to 5km did not have a large effect on model outputs, as is mentioned in the Discussion (with results presented in the ESM). Understanding what might happen when the patch size is changed is not straightforward because there are multiple consequences from doing this: fewer cases per patch (so reduced apparent persistence of infection), but also changes in the pattern of between-patch variation in the covariates.

***Reviewer #4****: While the subject of the study is of great interest and would benefit readers, more work is required on the presentation of the manuscript. Overall, the manuscript provides a mathematically robust and flexible framework for dealing with missing population data in spatial epidemiology.*

**Response**: Thanks.

*However, my initial impression is that the manuscript is too lengthy (25 pages of the manuscript and an additional 25 pages containing 10 appendices), which may divert readers' attention. The manuscript would greatly benefit from an effort by the authors to shorten the text, summarize the appendices within the main text, and integrate the explanation of the model’s application with case study and parameter estimation, rather than presenting them in separate sections.*

**Response**: We completely agree that the text of the main manuscript is quite lengthy, but we don’t believe that it can be substantially shortened without compromising some of the key messages of the manuscript. We do not see any merit in shortening supplementary materials (ESM) which provide interested readers with details critical to reproducing our work. However, we have moved some text to the ESM in order to streamline the presentation in the main text e.g. parts of the methods such as the presentation of the likelihood function. In addition, we have also added short summaries at the start of ‘Bayesian inference and prediction based on disease case reports’ in Methods (Page 6, lines 18-20) and Results (Page 10, lines 12-21) that give readers permission to skip these sections on initial reading. We believe that the case study is best presented as a separate section after the simulation study, and that trying to integrate it with other sections could confuse the reader.

*The text is logically coherent and maintains a consistent framework throughout, but the exposition could be streamlined for clarity. Most sections are dense and may be challenging for readers without a strong mathematical background. Simplification would benefit the overall readability. For example, the likelihood expression and Bayesian updates, while technically accurate, are dense and could overwhelm readers unfamiliar with Bayesian modeling or epidemic processes.*

**Response**: We agree that some parts of the presentation could be moved to the ESM in order to simplify the presentation. The Bayesian updates were already in the ESM (Appendix 3), but we have now also moved the likelihood expression and some other text to the ESM (Appendix 3).

*The paper would benefit from merging the explanation of the model with its application, both in the case study and the estimation of key parameters, rather than separating them into distinct sections. This would improve the flow and make it easier to understand the model’s real-world implications.*

**Response**: We respectfully disagree that merging the exposition of the model with its applications would improve the flow of the manuscript. One key reason is that we expect the framework to have applications beyond the scope of the analyses presented in the simulation study and the case study. Indeed, further clarifications on potential future applications of iPAR has been a recurring theme in the reviews we have received. For this reason, we feel that it is best to initially present the iPAR framework in a more general context – which shows at least some of its potential for other applications – before focusing on the more specific setting of the simulation study and case study.

*The manuscript demonstrates a flexible approach to addressing uncertainties in the spatial distribution of populations at risk. By relying on covariates, the model accommodates varying levels of data availability, making it adaptable to scenarios where detailed population data is missing. However, the effectiveness of this flexibility depends on the quality and relevance of the covariates used. For this study, only land use was employed as a covariate, but further discussion on the selection and justification of covariates could enhance the model's applicability.*

**Response**: We agree that a key strength of iPAR is its flexible approach to addressing uncertainties in the spatial distribution of populations at risk, but also that its effectiveness in practice is tied to the quality and relevance of the covariates used. To address this point we have added further detail in Methods on selection of a spatial resolution for the covariates (Page 4, lines 25-28). We have also added some detail in the discussion about using population density estimates as a covariate if they are available (Page 22, lines 20-25). ‘Benefits of modelling spatial variation in susceptibility and infectivity’ in Results can be seen as justification for using the land use covariates to model susceptibility and infectivity, in the sense that the model with the covariates performs better than the model without covariates.

*The methodology is mathematically specified with clear equations, but reproducibility in practice could depend on the following aspects:

Details on the implementation and parameter estimation, including the software used.
Explanation of how covariates are pre-processed, standardized, or selected (i.e., defining what constitutes a "suitable covariate").*

**Response**: We have made some edits to clarify the implementation – we’ve added details of the programming languages used (Page 7, lines 34-35). The github repository now contains documentation describing the software for simulation and inference, and scripts and example data files enabling interested readers to use the provided software more easily. Pre-processing of covariates consists of spatial aggregation to the selected patch size for the land use data used in the manuscript. We believe that the covariates are described in sufficient detail to enable reproducibility (Page 17, lines 34-40) – and in fact the covariates used in our analyses are those provided in the github repository as file covariates.dat. We did not use standardization for our covariates but can see situations where this might be helpful when using non-compositional covariates. Finally, we rephrased the penultimate paragraph of the Introduction, removing the phrase “suitable covariate” and instead adding more details on the selection of possible covariates (Page 3, lines 33-40). See also our response directly above this one.

*Simplification or rephrasing of the explanation and interpretation of susceptibility, infectivity, covariate-based functions, and parameters to make them more approachable.*

**Response**: We think that there’s limited scope for simplification. However, we have made some edits to the methods in places where the terminology was not clear e.g. we now use the term “non-compositional covariates” rather than “absolute covariates” because the former seems more standard (Page 5, lines 25-30). We also introduce the idea of the susceptibility and infectivity surfaces in the Introduction (Page 3, lines 13-23), at an intuitive level, which hopefully prepares the reader for the mathematically rigorous specification of the surfaces in the methods.

*While the incorporation of absolute and relative covariates is mathematically valid and aligns with common practices in modeling spatial heterogeneity, clarification is needed. For instance, what defines temperature as absolute and land cover as relative? If only land cover is used in the application, the entire explanation of absolute and relative covariates could be removed, keeping only the relevant covariate used in the model.
Clear explanations of what each parameter represents, the rationale behind the values or algorithms used to estimate them, and the references used to assign these values.*

**Response**: This is a good point, the terms ‘absolute’ and ‘relative’ are not at all standard – so we have changed the terminology to ‘non-compositional’ and ‘compositional’ respectively – with a reference added to a text on compositional data analysis (Page 5, lines 25-33). The explicit recognition of such compositional covariates is not something we have seen much of in the literature. We wanted to potentially allow for both compositional and non-compositional covariates – with a view to future applications of the framework - which is why we have retained both in the model formulation.

*The choice of non-informative priors requires further justification. Informative priors could be incorporated based on relevant epidemiological, spatial, and temporal factors. For example, proximity to known sources (infection time may depend on the distance from other infected patches), wild boar or farm density (higher densities may lead to earlier infections), patch-level biosecurity (better biosecurity could delay infection), seasonality (ASF transmission may exhibit seasonal trends), outbreak start time (infection times should logically follow the initial outbreak in nearby patches), and ASF's tendency to spread via contact (e.g., wild boar migration, human-mediated transmission). Additionally, models for spatial spread (e.g., diffusion or gravity models) could inform the selection of priors.*

**Response**: The ability to incorporate such prior information is indeed a key strength of Bayesian approaches such as iPAR. Thus prior information could be included if it was available, but in many cases it may not be available. Further, we have shown that it is not necessary in order to conduct an analysis. We also emphasise that the case study is intended to demonstrate iPAR in action, rather than an attempt to construct a definitive model for the spread of ASF.

*Other minor comments:

Acronyms should be spelled out the first time they are mentioned in the manuscript (e.g., MCMC). The term "ESM" can be removed and simply refer to the appendix number.
Incorporating line numbers would be a useful practice for facilitating specific revision comments.
Ensure that references are cited correctly in the text. For example, on page 6, it should read: O'Neill and Roberts (1999), Jewell et al. (2006), and Stockdale et al. (2017). Revise all reference citations accordingly.
When citing other authors, summarize their arguments clearly to provide context to the reader.*

**Response**: We now define all acronyms on first use. We have removed ‘ESM’ from the text and have incorporated line numbers – apologies for not providing these in the initial submission. The references will ultimately be reformatted according to the journal’s preferred style so we’ve kept the references as they are for the time being. Finally, we think that sufficient context has been provided when citing other publications. We could add additional context – this might be helpful to some readers but would also add more words to an already lengthy manuscript.

Thank you for welcoming a revision of our manuscript. We look forward to hearing from you.

Yours sincerely,

Stephen Catterall, PhD Mathematical Modeller

Biomathematics and Statistics Scotland

*On behalf of the authors*
